# Atomistic insights into the nucleation and growth of platinum on palladium nanocrystals

**DOI:** 10.1038/s41467-021-23290-x

**Published:** 2021-06-02

**Authors:** Wenpei Gao, Ahmed O. Elnabawy, Zachary D. Hood, Yifeng Shi, Xue Wang, Luke T. Roling, Xiaoqing Pan, Manos Mavrikakis, Younan Xia, Miaofang Chi

**Affiliations:** 1 Department of Materials Science and Engineering, North Carolina State University, Raleigh, NC USA; 2 Department of Materials Science and Engineering, University of California, Irvine, Irvine, CA USA; 3 Department of Chemical and Biological Engineering, University of Wisconsin - Madison, Madison, WI USA; 4 School of Chemistry and Biochemistry, Georgia Institute of Technology, Atlanta, GA USA; 5 School of Chemical and Biomolecular Engineering, Georgia Institute of Technology, Atlanta, GA USA; 6 Department of Physics and Astronomy, University of California, Irvine, Irvine, CA USA; 7 The Wallace H. Coulter Department of Biomedical Engineering, Georgia Institute of Technology and Emory University, Atlanta, GA USA; 8 Center for Nanophase Materials Sciences, Oak Ridge National Laboratory, Oak Ridge, TN USA; 9 Present Address: Chemical Engineering Department, Faculty of Engineering, Cairo University, Giza, Egypt; 10 Present Address: Department of Chemical and Biological Engineering, Iowa State University, Ames, IA USA

**Keywords:** Synthesis and processing, Characterization and analytical techniques, Nanoparticles

## Abstract

Despite the large number of reports on colloidal nanocrystals, very little is known about the mechanistic details in terms of nucleation and growth at the atomistic level. Taking bimetallic core-shell nanocrystals as an example, here we integrate in situ liquid-cell transmission electron microscopy with first-principles calculations to shed light on the atomistic details involved in the nucleation and growth of Pt on Pd cubic seeds. We elucidate the roles played by key synthesis parameters, including capping agent and precursor concentration, in controlling the nucleation site, diffusion path, and growth pattern of the Pt atoms. When the faces of a cubic seed are capped by Br^−^, Pt atoms preferentially nucleate from corners and then diffuse to edges and faces for the creation of a uniform shell. The diffusion does not occur until the Pt deposited at the corner has reached a threshold thickness. At a high concentration of the precursor, self-nucleation takes place and the Pt clusters then randomly attach to the surface of a seed for the formation of a non-uniform shell. These atomistic insights offer a general guideline for the rational synthesis of nanocrystals with diverse compositions, structures, shapes, and related properties.

## Abstract (web summary)

Creating predictable, controllable nanoparticles relies on a mechanistic understanding of their synthesis. Here, through integrated in situ liquid microscopy and first-principles calculations, the authors elucidate the atomistic details involved in the formation of colloidal core-shell nanoparticles.

## Introduction

Core-shell bimetallic nanocrystals have been actively explored as a new class of catalytic materials with enhanced activity, selectivity, and/or durability^1–7^. When the thickness of the shell is reduced down to a few atomic layers, they also offer a viable system for accomplishing cost-effective and sustainable use of scarce metals in catalysis by increasing their utilization efficiency^6,8–11^. Despite the successful use of seed-mediated growth for generating such nanocrystals, it remains a grand challenge to precisely control the products due to the lack of an atomic understanding of the nucleation and growth processes, as well as the explicit role played by each experimental parameter^12–14^. Attempts to generate an ultrathin, conformal, and uniform shell around the seed often ended up in the formation of poorly-defined islands on the surface. So far, the mechanistic understanding has been largely achieved using a quench-and-characterize approach^15^, where the synthesis is terminated at different time points to withdraw samples for electron microscopy analysis. Such an ex situ method inevitably leaves gaps between the samples, making it difficult to capture the mechanistic details involved in nucleation and growth. The recent advancement in liquid-cell transmission electron microscopy (LC-TEM) offers a great opportunity to address this long-standing issue. Most of the LC-TEM work reported in literature has focused on the formation of nanocrystals in a liquid phase through self-nucleation and attachment growth^16–23^. As limited by the poor resolution, it was only feasible to resolve small particles rather than individual atoms. By switching to heterogeneous nucleation in the presence of well-defined seeds, LC-TEM offers the ability to elucidate the atomistic details, including identification of the site for nucleation and the path for surface diffusion and growth.

Here we use in situ LC-TEM to uncover the mechanistic details involved in the formation of core-shell nanocrystals. Specifically, we focus on how Pt atoms nucleate and grow on the surface of Pd cubic seeds for the generation of Pd@Pt core-shell nanocubes. The experimental conditions are similar to what have been optimized for the batch synthesis^8,9,11,24^, except that the electrons for TEM imaging are used in place of the reducing agent. In addition, the experiments are conducted at room temperature to slow down the surface diffusion of Pt adatoms for tracking the growth process with atomic resolution. We systematically evaluate the roles of surface capping agent and precursor concentration in determining the site for nucleation and initial deposition, as well as the surface diffusion path and growth pattern. We also perform density functional theory (DFT) calculations to demonstrate how the capping agent influences the nucleation and diffusion of Pt atoms on various types of Pd facets^25–27^.

## Results

### Nucleation and growth of Pt in the presence of Br^−^ ions and at a low Pt^II^ precursor concentration

**Fig. 1:**
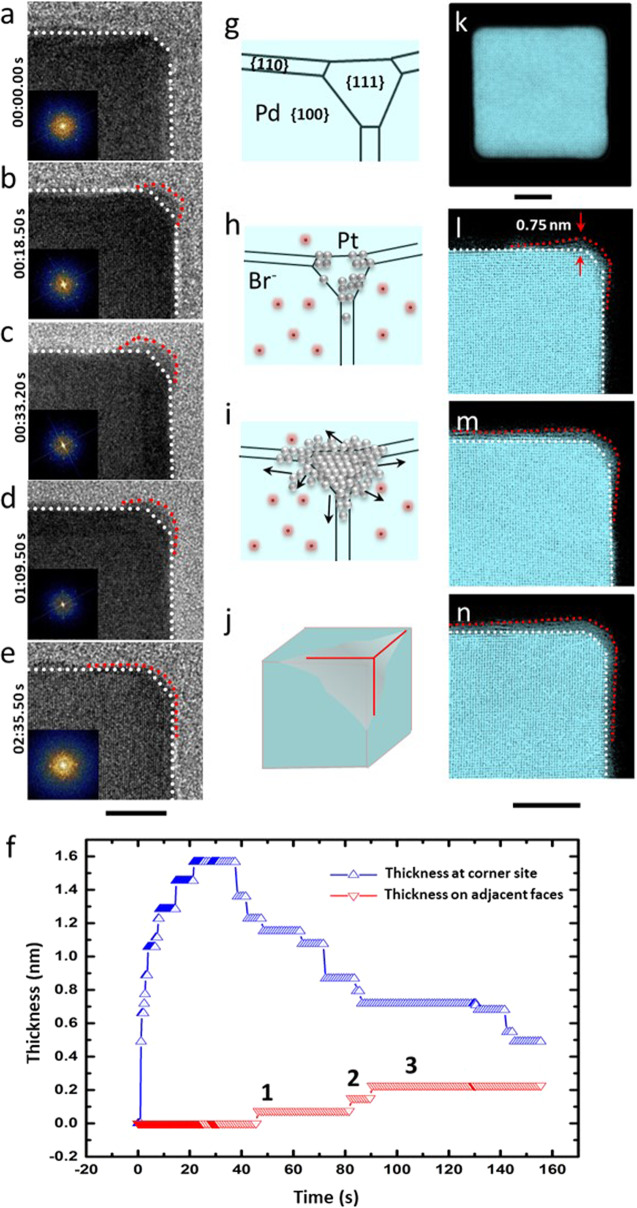
In situ observation of Pt deposition on Pd cubic seeds in the presence of Br^−^-capping for faces. **a**–**e** Time-elapsed in situ TEM images recorded from one of the corners of a Pd cubic seed (with FFT in the inset): **a** The cubic seed. **b** Initial deposition of Pt on the corner. **c** Continuous deposition of Pt on the corner. **d** Thinning of the Pt just deposited on the corner. **e** Formation of a conformal Pt shell. The white and red dotted lines indicate the projected profiles of Pd and Pt, respectively. **f** The thicknesses of Pt on the corner and adjacent face, respectively, as a function of time. **g**–**j** Schematics of the growth behavior of Pt on a Pd cubic seed in the presence of Br^−^-capping for faces. **k**–**n** Ex situ atomic-resolution HAADF-STEM images showing Pt deposition on the corner, followed by diffusion onto adjacent edges and faces. The scale bar under panel **e** applies to panels **a**–**e** and the scale bar under panel **n** applies to panels **l**–**n**. Scale bars: 5 nm.

The experimental setup used for the in situ LC-TEM study is schematically illustrated in Supplementary Fig. 1a. An aqueous mixture containing Pd cubic seeds (20 nm in edge length) and a specific amount of K_2_PtCl_4_ was sealed in the space between two SiN windows (*ca*. 40 nm thick) that are transparent to the electrons used for TEM imaging. The protocol is similar to what is typically used for a batch synthesis (Supplementary Fig. 1b), where Pd cubic seeds are mixed with a reducing agent in a container and a Pt^II^ precursor is slowly titrated into the mixture using a syringe pump^24^. For the batch synthesis, a reducing agent has to be introduced in order to convert the Pt^II^ precursor to its elemental form. In the in situ experiment, the electrons used for imaging play the dominant role as a reducing agent and those precursor molecules exposed to the electrons will be reduced. The Pd cubic seed was primarily enclosed by six faces in the form of {100} facets, as confirmed by the TEM image and corresponding FFT pattern in Fig. 1a. The minor truncation led to the exposure of {110} and {111} facets at small fractions on the surface as edges and corners, respectively. In designing the experiments, we aimed to elucidate the roles played by Br^−^ ions (a capping agent for the Pd{100} facets) and the concentration of Pt^II^ precursor in determining the nucleation and growth of Pt on Pd cubic seeds. As summarized in Supplementary Fig. 2, four combinations of reaction conditions were evaluated, with the Pt^II^ precursor being used at two different concentrations in the presence/absence of face-capping by Br^−^ ions.

We intentionally used the Pt^II^ precursor at a relatively small amount to ensure that the resultant Pt atoms would be inadequate to cover the entire surface of the Pd seed. Such a condition allowed us to capture the dynamics of atom diffusion. Figure 1b–e shows a set of in situ TEM images recorded from the corner region of the same Pd cubic seed during the nucleation and growth of Pt in the presence of Br^−^ ions and at a relatively low concentration of 0.015 mM for the Pt^II^ precursor. It should be pointed out that the Br^−^ ions not only served as a capping agent for the Pd{100} facets but also significantly slowed down the reduction rate of the Pt^II^ precursor through ligand exchange (*PtCl*_*4*_^*2−*^ *+ 4Br*^*−*^ *→ PtBr*_*4*_^*2−*^ *+ 4Cl*^*−*^)^28^. The continuous trajectory of nucleation and growth can be found in Supplementary Movie 1. As marked by the red dotted lines in Fig. 1b, the Pt atoms preferentially nucleated and then grew from the corner when the Pt^II^ precursor was reduced by the solvated electrons. At *t* = 18.5 s, the Pt deposited on the corner reached a thickness of 1.5 nm, generating a protruding structure on the Pd{111} facet. The packing of the Pt atoms followed that of the Pd atoms in the seed, with an epitaxial relationship of Pt(100)[010]||Pd(100)[010]. When the thickness of the Pt deposited on the corner reached a critical value of *ca*. 1.6 nm at *t* = 33.2 s (Fig. 1c), some of the Pt atoms started to diffuse to adjacent edges and then faces. It should be pointed out that different corners may have different degrees of truncation, and thus different areas for the {111} facets. As a result, it is not uncommon to observe different threshold values needed for triggering the diffusion of Pt atoms away from the Pt overlayers deposited on different corners. As shown by the initial frame of Supplementary Movie 1, the bottom left corner was more truncated than the top right corner, so the Pt atoms deposited on the bottom left corner did not diffuse out after the overlayers had reached a thickness similar to those at the top right corner. As shown in Fig. 1d, e for two additional time points, the thickness of the Pt deposited on the corner further decreased while those on the adjacent faces increased, generating a thin, uniform, conformal coating on the Pd cubic seed.

The in situ observation of surface diffusion from corners to edges and faces was consistent with our current understanding based upon ex situ TEM studies^24^. More significantly, it revealed, for the first time, that such diffusion would not occur until the initially deposited Pt reached a threshold thickness. We also quantitatively analyzed the evolution of Pt thickness on the corner and adjacent face as a function of time (Fig. 1f). The thickness at the corner increased at an average growth rate of 0.08 nm/s over the first 20 s, with the initial stage favoring a much faster growth rate. The difference in thickness between *t* = 8.4 s (1.28 nm) and *t* = 14.7 s (1.45 nm) reflects the buildup of one atomic layer. The overlayer at the corner reached a thickness of 1.57 nm at *t* = 21.6 s, corresponding to 9 layers of Pt atoms. From *t* = 40 s to 160 s, the Pt overlayer at the corner decreased discretely in thickness whereas the thickness of Pt on one of the adjacent faces increased to 0.3 nm. The moment when Pt was seen to accumulate on the faces (at time points 1, 2, 3 labeled in Fig. 1f) coincided with the decrease in Pt thickness at the corner except for the delay of several seconds. A similar observation was also made in a separate experiment conducted under the same conditions (Supplementary Fig. 3). Figure 1g–j shows a schematic illustration of the growth behavior, including the initial nucleation and growth at the corner, followed by directional diffusion of Pt adatoms from the corner to adjacent edges and faces.

Our in situ observation was also in agreement with the data from an ex situ study. One of the advantages of using electrons as a reducing agent is that we can quickly terminate the reduction of the Pt^II^ precursor by moving away the electron beam. In this case, we can quench the reaction in the liquid cell at different stages of a synthesis and sample the nanocrystals for ex situ analysis, with a purpose of achieving atomic resolution. Figure 1k–n shows the ex situ high-angle annular dark-field scanning transmission electron microscopy (HAADF-STEM) images recorded from four different nanocrystals that cover the stages shown in Fig. 1a–e. The image in Fig. 1k confirms the presence of atomically-flat faces on the Pd cubic seed. The corner of the cubic seed showed a truncation of approximately 5 atomic layers from the projection of {100}. As depicted by the model in Fig. 1g, the surface of the cubic seed also contained {110} and {111} facets as the edges and corners, respectively. Figure 1l indicates that the initial deposition of fewer than three atomic layers (0.75 nm) took place at the corner, together with a smooth transition into the adjacent faces, as marked by the red outline. The Pt on the face showed a larger plane spacing of 0.246 nm between the adjacent layers parallel to the Pd surface, in contrast to 0.195 nm of bulk Pd and 0.196 nm of bulk Pt (Supplementary Fig. 4). Such expansion was likely caused by the collective effects of the in-plane surface compression arising from the epitaxial core-shell structure and the adsorption of Br^−^ ions to the outmost surface^29^.

It should be pointed out that the darker contrast of Pt on the corner in Fig. 1m, n, as compared to the brighter contrast of Pd, can be ascribed to the small amount of Pt on the Pd cubic seed. This was confirmed by high-resolution HAADF imaging, where the contrast of the Pt overlayer was lower than that of the Pd core, indicating a thinner thickness for the Pt atoms relative to that of the Pd core along the beam direction (Supplementary Fig. 4). A comparison of the experimental image with the simulated HAADF images with different numbers of Pt atoms along the beam direction reveals that the diffusion of the Pt adatoms from the edge to the face extended over a distance of about 20 atoms (Supplementary Figs. 5–9, and the related discussion in Supplementary Information). The higher contrast for the first Pt atomic layer (the one interfacing with the Pd surface) relative to those of the outer Pt atomic layers (Supplementary Fig. 4) indicates that the coverage of Pt atoms decreased with the distance away from the Pd surface.

### Nucleation and growth of Pt in the absence of Br^−^ ions and at a low Pt^II^ precursor concentration

**Fig. 2:**
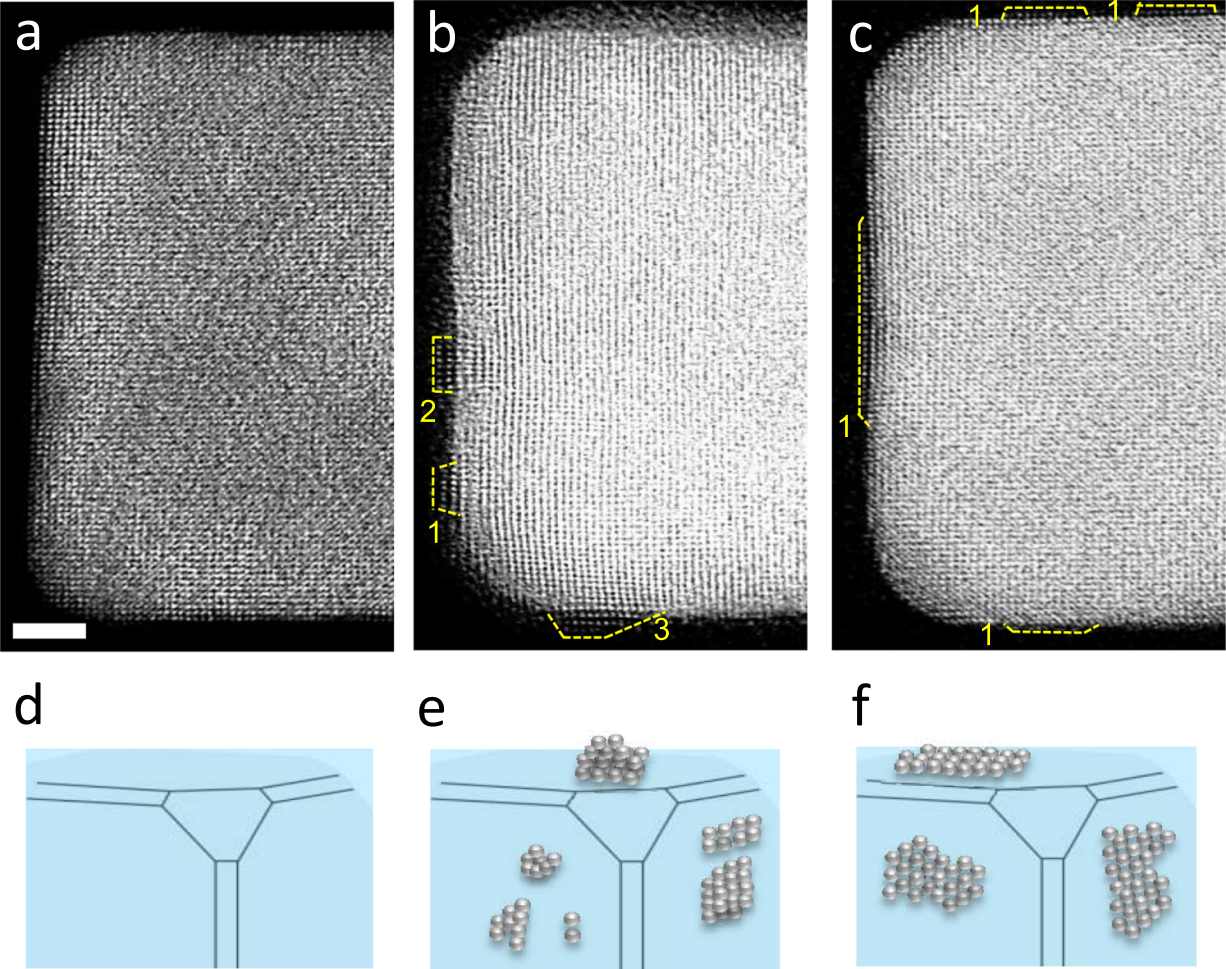
Ex situ observation of Pt deposition on a Pd cubic seed in the absence of Br^−^ ions. **a** Part of the original Pd cubic seed. **b** Part of a different seed with Pt islands of 1–3 atomic layers in thickness on the faces. The Pd seed was under electron beam illumination for 50 s in the liquid cell. **c** Part of a third seed with relatively large Pt islands of one and two atomic layers in thickness on the faces. The Pd seed was under electron beam illumination for 150 s in the liquid cell. The yellow dashed line outlines the deposited Pt and the number next to the line indicates the thickness of the island (in atomic layers). **d**–**f** Schematics of the growth trajectory of Pt on a Pd cubic seed in the absence of Br^−^ ions. Scale bar: 2 nm.

In an effort to elucidate the effect of Br^−^ ions on the nucleation and growth of Pt on Pd cubic seeds, control experiments were conducted under the same conditions except that no KBr was added into the liquid cell. Based on the local contrast in STEM images (Fig. 2), the deposition of Pt was found to occur mainly on the faces, rather than the corners or edges. Specifically, small Pt islands of 1-3 atomic layers in thickness (with a majority of two atomic layers) were observed on scattered locations on the faces. The thickness of the island along the beam direction was too thin to generate enough contrast during in situ studies. As such, we could only obtain atomic-resolution images under ex situ conditions using the method described for Fig. 1k–n. By benchmarking against the simulated images (Supplementary Fig. 8), the Pt islands shown in Fig. 2 contained 7-11 Pt atoms along the beam direction, confirming that the Pt islands were situated on faces rather than edges. As shown in Fig. 2c, some of the Pt islands could be as thin as one atomic layer in thickness and span across a relatively large area. Such a Pt monolayer was likely formed from the islands of 2–3 layers in thickness (see, Fig. 2b) through surface diffusion. In contrast to the case involving KBr, the Pt atoms tended to nucleate and grow from faces rather than corners due to the higher surface energy and larger area associated with the faces^28^. Some Pt atoms could also nucleate and grow from the edges due to their higher surface energy relative to both faces and corners, but the probability was limited by their small area. The random formation of Pt islands on the faces can also be attributed to the increase in reduction kinetics for the Pt^II^ precursor because of the absence of Br^−^ ions in the reaction system^30^.

### Effects of precursor concentration on the nucleation and growth mechanisms

**Fig. 3:**
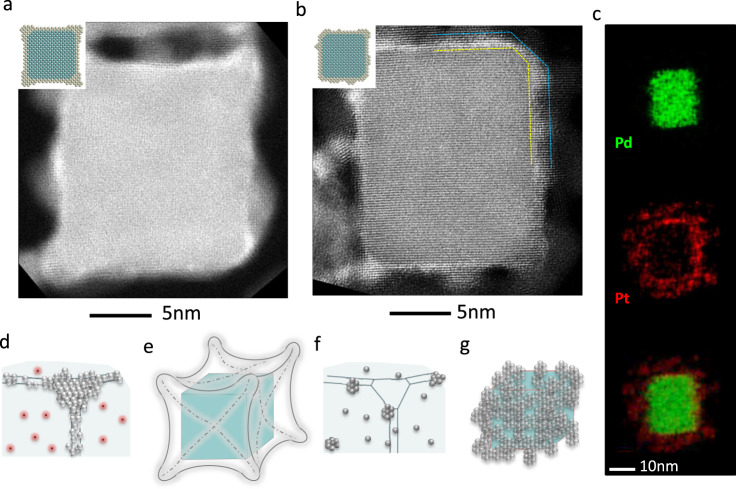
Deposition of Pt on Pd cubic seeds at a high concentration for the precursor. **a** In the presence of Br^−^ capping on faces, Pt preferentially grows from corners, generating a Pd@Pt concave nanocube. The sample was under electron beam illumination for 40 s in the liquid cell. **b** In the absence of Br^−^ capping, Pt grows randomly from the surface. The sample was illuminated by electron beam for 90 s in the liquid cell. **c** EDS mapping of the nanocube in **b**. **d**–**g** Schematics of the growth trajectory of Pt on a Pd cubic seed in the (**d**, **e**) presence and (**f**, **g**) absence of Br^−^ ions, at a much higher concentration for the precursor than the study shown in Fig. 1.

The use of the Pt^II^ precursor at a low concentration of 0.015 mM allowed us to gain insights into the atomistic details involved in the nucleation and growth of Pt atoms on Pd cubic seeds. In a batch synthesis, however, the Pt^II^ precursor is typically used at a much higher concentration in order to form a complete shell on the seed. This is also the reaction condition under which poorly-controlled products tend to appear, including the creation of Pt shells non-uniform in thickness and/or rough in outer surface, and the formation of Pt nanoparticles due to self-nucleation. To this end, we used in situ LC-TEM to investigate the growth of Pt on Pd cubic seeds in the presence of KBr but at a much higher (1.5 vs. 0.015 mM) concentration for the Pt^II^ precursor. The higher concentration resulted in faster reduction kinetics and thus quicker deposition of Pt atoms at the corners for the creation of a concave structure (Supplementary Movie 2 and Supplementary Fig. 10). Figure 3a shows the HAADF-STEM image of a representative nanocrystal sampled from the liquid cell. The Pt shell took a concave structure, with more significant growth at the corners than both edges and faces. Based on EDX analysis (Supplementary Fig. 11), the Pt shell covered the entire surface of the seed, as illustrated by the atomic model in the inset. Small Pt islands were also observed on the faces of the seed, but their formation should be attributed to the attachment of clusters formed in the solution via self-nucleation as a result of the high concentration of the Pt^II^ precursor^16^. The islands tended to form randomly on the area illuminated by the electron beam, albeit the presence of Br^−^ ions on the faces made the corners and edges more favorable for the attachment of clusters. Some of the clusters could also be deposited on the faces, but on top of the Br^−^ capping layer, as revealed by the white gap between the Pd seed and the Pt islands (Supplementary Fig. 10a–d and Supplementary Fig. 11). The growth rate of Pt at the corner was measured to be 0.7 nm/s, almost 10 times faster than the case shown in Fig. 1, confirming that the reduction kinetics and thus growth rate had a strong dependence on the concentration of the Pt^II^ precursor. No measurable growth was detected on the faces for the first 30 s, again suggesting that the diffusion of Pt from corners to edges and faces only took place after the build-up of Pt at the corner to a certain thickness, regardless of the precursor concentration.

In the absence of Br^−^, a high precursor concentration resulted in a relatively thick Pt shell on Pd seed, as shown by the real-time video in Supplementary Movie 3, snapshots in Supplementary Fig. 12, and EDS mapping data in Fig. 3c. Compared to the case with a low precursor concentration in the presence of Br^−^, the shell growth here occurred primarily via the initial formation of Pt clusters in the liquid, followed by their attachment to the faces, adhesion, re-orientation, and coalescence through a non-classical epitaxial growth mechanism. This growth behavior is clearly evidenced at the atomic scale by high-resolution STEM imaging (Supplementary Fig. 13). The dynamic size change of the core-shell particle as a function of synthesis time is plotted in Supplementary Fig. 12d, giving a growth rate of 0.013 nm/s. The attachment of Pt clusters over the entire surface led to the formation of a non-uniform Pt shell, most likely due to the inadequate diffusion of Pt clusters across the surface.

To understand the results from an in situ LC-TEM study and thus their implications for a batch synthesis, it is important to note their similarities and differences in terms of both reduction mechanism and kinetics. Compared with the chemical reductant typically used in a batch synthesis, the strong reducing power of the electron beam, including the H• radical derived from electrons in an in situ experiment can lead to quick, almost instantaneous reduction of the precursor^31–33^. Despite the difference in reducing power, the rate of metal deposition is also proportional to the concentrations of both the precursor and reductant. In designing the LC-TEM experiments, we have considered this aspect to ensure that the observations made in a liquid cell can be directly compared with the results of a typical batch synthesis. Specially, for the LC-TEM study described in Fig. 1, we purposely reduced the concentration of the precursor while using a small dose of beam irradiation to ensure that the reduction rate and thus metal deposition rate would be on par with those involved in a typical batch synthesis. In another set of experiments described in Fig. 3 and Supplementary Fig. 10, we increased the precursor concentration to a level similar to that commonly used in a batch synthesis so the reduction rate would become much greater. In this case, Pt clusters quickly formed in the solution under the same beam condition, even in areas away from the Pd seeds, as a result of the significantly increased reduction rate and thus the involvement of self-nucleation. It is worth pointing out that the growth behaviors observed in the LC-TEM experiments, including the morphology of the products, were all observed in the setting of batch synthesis.

### Nucleation and diffusion mechanisms interpreted by DFT calculations

To gain an understanding of the dynamics of nucleation and diffusion of Pt on the Pd surface observed in the experiments, we performed DFT calculations on a structurally-simple corner model as shown in Supplementary Fig. 14. The corner model is representative of the Pd cubic seed involved in the experiment (Fig. 1 and Supplementary Fig. 1c) as it displays {100}, {110}, and {111} facets as the faces, edges, and corners, respectively. In the case with Br covering the {100} faces of a Pd cubic seed, a coverage of 0.5 monolayer (ML) was identified as the most relevant structure to compare with the experimental observations (see Supplementary Figs. 15 and 16, and Supplementary Information). We would like to note a few limitations of our model. First, we do not account for the solid-liquid interface surrounding the Pd nanocubes, which prohibits us from commenting on such phenomena as the reduction of the Pt^II^ precursor, deposition of the Pt atoms unto the Pd seeds, or self-nucleation of Pt clusters in the solution and their attachment to the Pd nanocubes. Additionally, we focus on the case involving a low concentration of the precursor since it can help us understand nucleation and diffusion of Pt on the Pd corner model without involving cluster formation. Finally, we do not include thermal effects for the diffusion pathways investigated here. Notwithstanding those limitations, the model we study here makes a good compromise between simplicity and comprehensiveness by focusing on the energetics of diffusion of the deposited Pt atoms on the Pd corner model and the effect of the Br adlayer on the diffusion process.

As shown by the results from in situ experiments, the absence or presence of Br will affect the site of nucleation for Pt. This is also supported by the calculation results. Supplementary Fig. 17 shows the relative stability of a Pt adatom on the Pd corner model, in the absence or presence of Br adlayer on the faces. In both cases, Pt strongly favors the Pd{100} facet to the Pd{111} facet. This explains the tendency of Pt to deposit on the faces of a cubic seed in the absence of Br. In the presence of Br, however, Pt only finds the {111} corners available for deposition even though it still energetically favors the Pd{100} faces.

According to the rationale illustrated above, in our calculations where the Br is present on the Pd{100} facets, we placed a 1 ML of Pt on the {111} corner of a Pd cubic seed and then calculated the possible diffusion paths for the Pt adatoms out of this ML. The same diffusion paths in the hypothetical scenario, where the Br adlayer was absent from the faces, were also examined to elucidate the effect of Br on Pt diffusion. Supplementary Fig. 18 depicts the various diffusion paths and their corresponding activation and diffusion energies, with the activation energy referring to the energy difference between the transition state along the diffusion path and the initial state, and the diffusion energy corresponding to the energy difference between the final state and the initial state in the diffusion elementary step. All the diffusion paths are highly activated regardless of the presence or absence of Br. The path via a substitution site (0 → a → 1) requires a much lower energy compared to the direct hopping of a Pt adatom from the corner to the {100} face, suggesting that the {110} edges likely play an important role in the spillover of the Pt thin layer from the corners of the cubic seed to its faces, consistent with the experimental observations.

## Discussion

The experimental observation that the Pt adatoms on the corners would not diffuse to edges and faces until their protrusion had a minimum thickness was also explained by calculations. To this end, we investigated the diffusion behavior of a single Pt adatom on a Pt layer (shortened as Pt adatom/monolayer in our discussion), compared to the diffusion of an atom out of a complete Pt layer. The Pt adatom/monolayer was adapted as a simplified model of the protrusion to keep the computational expense tractable while providing representative insights. Supplementary Fig. 19 depicts the various substitution diffusion paths available for the Pt adatom, both in the presence and absence of a Br adlayer on the faces. A comparison of the energies in Supplementary Figs. 18 and 19 reveals that the diffusion processes are generally more energetically driven in the case of Pt adatom/monolayer (i.e., diffusion energies were mildly endothermic or even exothermic) as compared to the diffusion of an atom out of a complete Pt layer (i.e., more endothermic diffusion energies). In addition, most of the diffusion activation energies were much lower in the case of Pt adatom/monolayer, relative to a complete Pt layer.

**Fig. 4:**
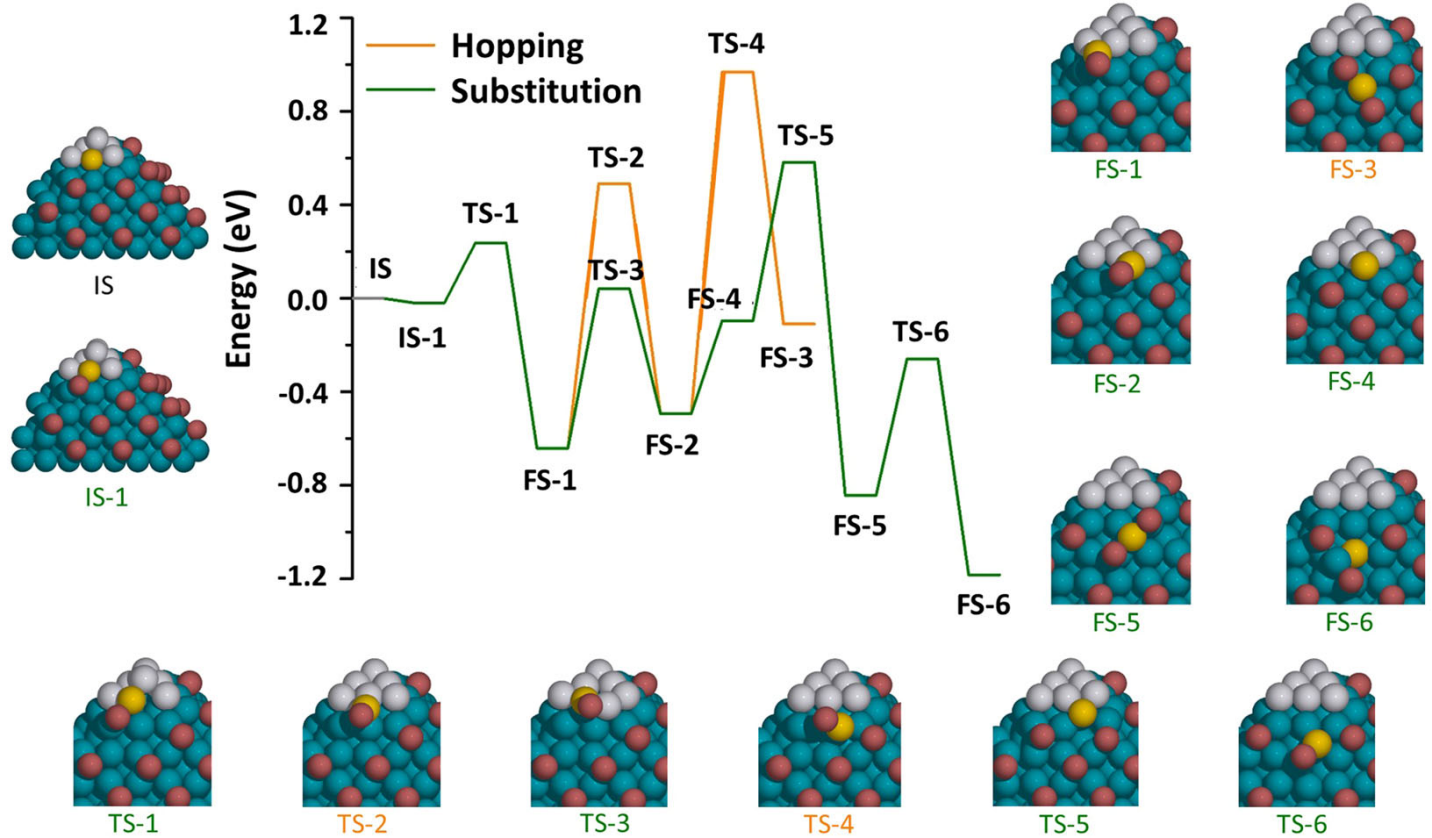
Potential energy diagram for the diffusion processes of a Pt adatom atop a Pt ML on the {111} corner. The hopping mechanism is represented with an orange line, while the substitution mechanism is represented by a green line. IS, TS, and FS stand for initial, transition, and final states, respectively. The structures of these states are represented by the insets surrounding the diagram. The labels of the insets are colored in orange if they belong to the hopping mechanism, or green if they belong to the substitution mechanism or are common among the two mechanisms, according to the legend of the potential energy diagram. In the insets, gray, blue, and red spheres represent Pt, Pd, and Br atoms, respectively. The yellow sphere is the diffusing Pt atom. Notice that FS-2 is common between TS-2 and TS-3, and therefore the yellow and the substituted gray spheres in TS-3 will appear as if they were exchanged in FS-2.

It is worth pointing out that the activation energies for most diffusion paths were higher in the presence of Br than in its absence (Supplementary Fig. 19), indicating a hindrance effect of the Br adlayer on the spillover of Pt from the corner to adjacent faces. The only two paths that escape such hindrance (i.e., that are easier in the presence of Br than in its absence) are those in which the Pt adatom displaces a Pt atom from the layer beneath into a {100} hollow site at the interface between the Pd face and the Pt layer at the corner (see “0 → 0 → 2” and “0 → 0 → 3” in Supplementary Fig. 19). The diffusion process of “0 → 0 → 3”, the easiest of all diffusion paths, is depicted in detail in Fig. 4. Starting from the initial state (IS), a minor reconstruction of the Br adlayer brings a Br atom into coordination with the Pd-Pt interface (IS-1). The Pt adatom then substitutes into the Pt ML beneath, yielding—through a transition state (TS-1)—a final state in which a Pt-Br complex is coordinated to the Pt-Pd interface (FS-1). These results also explain the experimental observation in Fig. 1, where the Pt spillover to the side faces could only take place after a substantial buildup of Pt at the corner. Following FS-1, Pt diffusion from corner to faces could follow two different directions: along or away from the Pd-Pt interface (Fig. 4). The activation and diffusion energies of the potential pathways associated with these two directions are described in Supplementary Figs. 20 and 21. While both directions of diffusion are highly activated, the relatively easier route is to diffuse along the Pd-Pt interface by substituting a Pt atom in the Pt layer (TS-3) and pushing it to a {100} hollow site at the Pd-Pt interface (FS-2) with an activation energy barrier of 0.68 eV (*i.e*., the difference between TS-3 and FS-1 in Fig. 4). In contrast, the hopping mechanism overall takes a relatively higher activation energy barrier of 1.13 eV (*i.e*., the difference between TS-2 and FS-1). Further diffusion away from the corners is shown in Fig. 4 and is discussed in detail in the Supplementary Information.

**Fig. 5:**
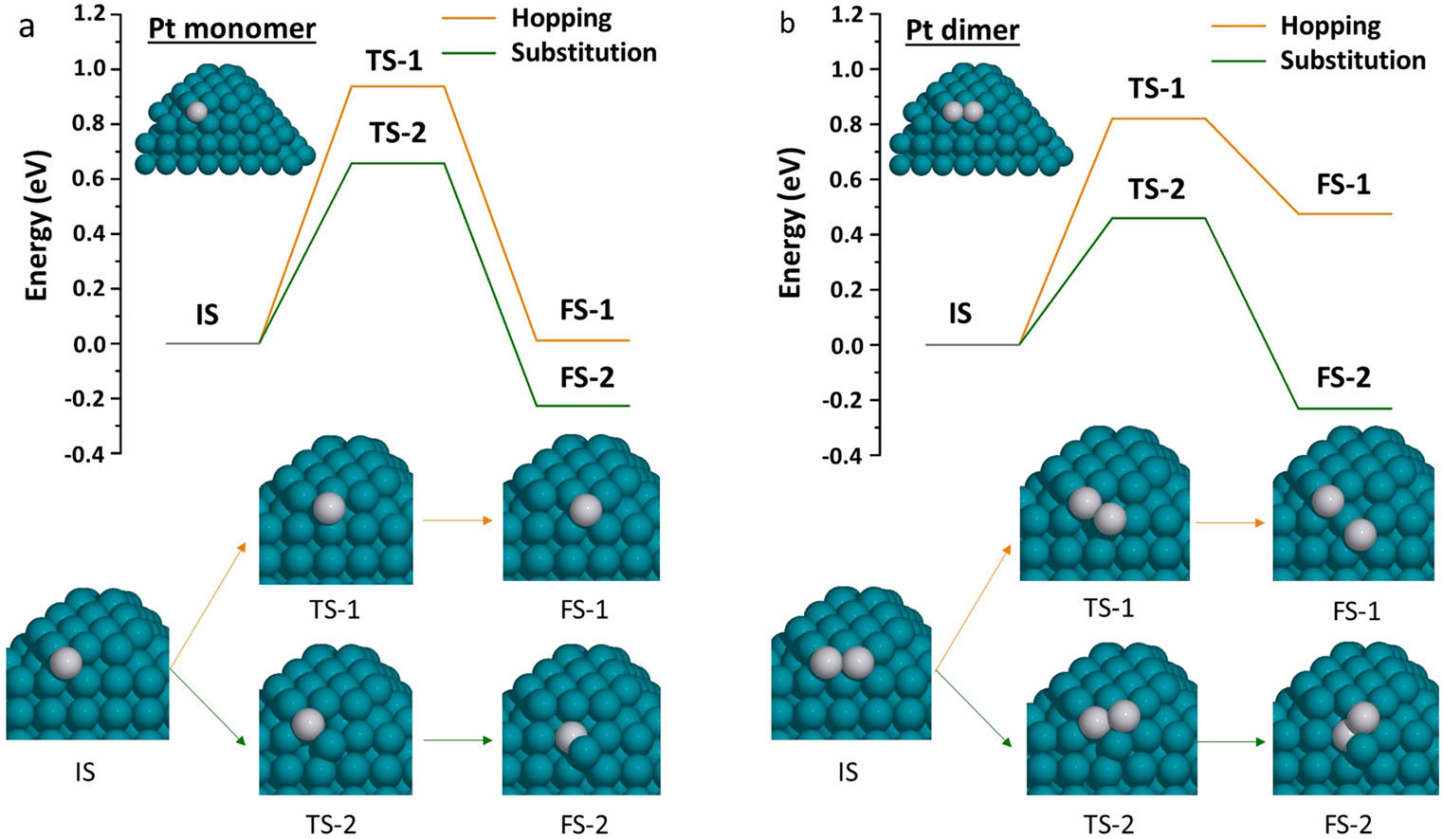
Potential energy diagrams for the easiest diffusion processes of Pt adatom on the {100} face. We examined the deposited Pt as **a** a monomer and **b** a dimer, respectively, on a Pd{100} face without Br adlayer. In either panel, the hopping mechanism is represented by an orange line, while the substitution mechanism is represented by a green line. IS, TS, and FS stand for initial, transition, and final states, respectively. The structures of these states are represented by the insets below each diagram. The insets representing the hopping mechanism are connected through orange lines, while the insets representing the substitution mechanism are connected through green lines, according to the legend of the potential energy diagrams. In the insets, gray and blue spheres represent Pt and Pd atoms, respectively.

In the absence of Br, Pt atoms preferentially adsorb onto the Pd{100} faces of the Pd seed, starting from a single Pt atom and going up to eight Pt atoms (enough to occupy all hollow sites available for adsorption in the unit cell; see Supplementary Information for details). The most favorable adsorption sites and configurations of these Pt atoms are shown in Supplementary Fig. 22. When a single Pt atom is deposited on a hollow Pd{100} site, the Pt atom can hop to other hollow sites over Pd-Pd bridge sites, or substitute an underlying Pd atom, pushing it to an adjacent Pd surface hollow site. The easiest hopping and substitution diffusion paths are shown in Fig. 5a, and all paths considered are shown in Supplementary Figs. 23 and 24. Our results indicate that the substitution mechanism is preferred for the diffusion of a Pt adatom on the Pd{100} face. In this path, the substituted Pd atom is displaced to an adjacent hollow site that is inaccessible to the Pt atom in a single hopping step. The diffusion energies calculated here agree well with our recent calculations on extended surface models^10,34^. Substitution was also found to be the preferred pathway for the case of a Pt dimer adsorbed on Pd{100}, as shown in Fig. 5b and Supplementary Fig. 25.

The formation of two-layer islands observed experimentally (Fig. 2, in the absence of KBr) can also be explained by calculations. As shown in Supplementary Figs. 22 and 26, both Pt pentamers and octamers favor the bi-layer island structure. The activation energy for breaking the two-layer structure was calculated to be at least 0.15 eV higher in the case of an octamer relative to that of a pentamer (Supplementary Fig. 26), indicating that islands with a relatively larger lateral size are energetically more preferred. This is consistent with the experimental observation, where all islands were larger than 5 atoms in width. In fact, the formation of two-layer islands is also supported by the fact that Pt adatoms prefer to coordinate with each other as opposed to being scattered on the Pd(100) face (Supplementary Fig. 22). Any Pt atoms or clusters on the Pd surface can serve as adsorption sites for the incoming Pt atoms, resulting in lateral growth into islands.

In summary, we have elucidated the atomistic details involved in the formation of Pd@Pt core-shell nanocubes. Our results indicate that the creation of a conformal, uniform shell critically relies on the diffusion of Pt adatoms from corners to edges and faces, and no diffusion will occur until a threshold thickness has been reached. Experimentally, such a growth pattern can be achieved through the use of a low concentration for the precursor and the addition of a capping agent for the faces. At a high concentration of the precursor, the growth is dominated by the attachment of Pt clusters formed via self-nucleation, leading to the formation of a non-uniform shell. Since the Pd seeds used in this work are enclosed by all three low-index facets, the insights into nucleation, diffusion, and growth should be extendible to metal nanocrystals with other compositions and shapes, allowing the rational development of their synthetic protocols.

## Methods

### Synthesis of the Pd cubic seeds

The Pd nanocubes were synthesized according to our previous report^35^. A mixture of PVP (105 mg, Aldrich, *M*_w_ ≈ 55,000), ascorbic acid (60 mg, Aldrich), and KBr (600 mg, Aldrich) were dissolved under magnetic stirring in a 20-mL glass vial containing 8 mL of deionized water. The mixture was then heated to 80 °C for 10 min in air. Next, 3 mL of an aqueous solution containing 57.0 mg of Na_2_PdCl_4_ (Aldrich) were added using a glass pipette. The vial was then capped and heated at 80 °C for 3 h. The solid product was collected by centrifugation and washed with deionized water ten times to obtain Pd cubic seeds with essentially no Br^−^ capping for the faces.

### Electron microscopy

In situ LC-TEM observation was carried out on an FEI Titan microscope operated at 300 kV using a SiN chip-based liquid cell holder (Protochips Poseidon 510). During the operation, an electron beam with a spot size of 200 nm in diameter was used, corresponding to a current density of 2 pA/cm^2^ on the screen. Upon assembly, 1.5 μL of the suspension of Pd cubic seeds (2 mg/mL) was added into the liquid cell. The Pd cubic seeds were firstly dispersed in deionized (DI) water, sonicated, and dispersed on the bottom chip, followed by drying to enhance their adhesion to the window. Afterwards, 0.1 µL of an aqueous Pt^II^ precursor solution (K_2_PtCl_4_, 0.015 mM, Aldrich) was added. A top chip was placed on the bottom chip to form a static cell with liquid inside. For the experiment involving Br^−^ capping, the initial concentration of Br^−^ ions in the cell was kept at 45.4 mM, while the Pt^II^ precursor was used at 0.015 and 1.5 mM, respectively. The concentration of the Pt^II^ precursor in the cell should be approximately the same as the stock solution, but possible drying of the droplet could make the sample more concentrated.

The involvement of a limited amount of liquid inside the cell ensured the formation of a thin layer of solution. When combined with the micro-well design of the SiN window, the electron scattering from both the liquid and window was minimized. Because the imaging electrons could reduce the precursor, no other reducing agent was added into the cell. TEM images were recorded using a Gatan OneView camera. The entire process was recorded as a series of bright-field TEM micrographs at a speed of 25 frame per second (FPS) with 4k × 4k pixels per frame. The sequential images were aligned to improve signal/noise ratio for further analysis. During the entire experiment, the nanocrystals only moved a few nm in distance and small in-plane rotations were occasionally observed. These movements were corrected in our distance measurements. Experimentally, we adjusted the focus every time before acquiring a TEM micrograph and it was estimated that the error caused by the movement of particles should not exceed 3 pixels or more than 0.4 nm in all measurements. High-resolution STEM micrographs were acquired using an FEI Titan STEM with a CEOS Cs probe corrector, operated at 300 kV.

### Density functional theory calculations

Periodic density functional theory (DFT) calculations were performed with the projector augmented wave (PAW) potentials^36,37^ and the generalized gradient approximation (GGA-PW91) exchange correlation functional^25^, as implemented in the Vienna ab initio Simulation Package (VASP)^26,27^. The electron wave function was expanded in plane waves truncated at a kinetic energy cutoff of 400 eV. The bulk lattice constant of Pd was calculated to be 3.95 Å, close to the experimental value of 3.89 Å^38,39^. Geometric optimization was performed until the Hellmann-Feynman forces on all atoms were below 0.02 eV/Å. Adsorption was allowed only on the relaxed surface of the slab, and the electrostatic potential was corrected for the dipole moment accordingly^40,41^.

The bromine (Br) adlayer structure on Pd(100) was calculated on a (4×4) unit cell of a four-layer metal slab, in which the bottom two layers were fixed at the optimized bulk positions, while the top two layers, as well as the Br adlayer, were allowed to fully relax. Vertical images of the slab were separated by approximately 24 Å, ensuring minimal interaction between images in the z-direction. The surface Brillouin zone was sampled with a 2×2×1 Monkhorst-Pack k-point mesh^42^; convergence of relative energetics with respect to k-point mesh was ensured.

A Pd cube in a (10×10) unit cell was selectively truncated to model a cubic corner composed of two {100} facets (composed of blue spheres in Supplementary Fig. 14a); the atoms making up the third {100} facet were removed for computational expediency. Atoms in the interior of the cubic corner model, as well as the atoms exposed at the truncated face, were fixed at their optimized bulk positions. The fixed atoms are represented by red spheres in Supplementary Fig. 14a; adsorption and diffusion of Pt atoms were avoided as much as possible on these fixed Pd atoms to prevent unrealistic energetics for the interaction between Pt adatoms and the underlying Pd atoms unable to move to accommodate (or be substituted by) the diffusing Pt adatoms. We further removed a row of edge atoms between the two {100} facets to expose the {110} facet (composed of green spheres in Supplementary Fig. 14a), as well as the corner atoms to expose the {111} facet (composed of yellow spheres in Supplementary Fig. 14a) characteristic of a truncated cubic corner. Supplementary Fig. 14b shows the most stable structure of a relaxed 0.5 ML Br adlayer. The bottommost Br atoms, when allowed to fully relax, tended to move and adsorb below the cubic model. To avoid this unphysical artifact, we fixed the z-coordinate of those Br atoms, while allowing the relaxation in the x and y coordinates. The Brillouin zone was sampled at the gamma point.

Minimum energy paths and metal atom diffusion barriers on the Pd corner model at 0.0 and 0.5 ML Br coverage were calculated using the climbing image nudged elastic band method^43^. The initial and final states were interpolated with seven intermediate images, each converged to 0.05 eV/Å maximum force on each atom (the calculated barriers were not found to vary at more strict criterion). Overall, at least 12 Å of vacuum was introduced to separate the adsorbed atoms from the periodic images in the lateral or vertical directions. In fact, our calculated binding energy of Pt in its most stable adsorption hollow site on the Pd(100) face (in the absence of Br) was nearly identical to what was previously calculated on a (3×3) extended surface of Pd(100)^34^, at −4.71 eV (on the corner model) and −4.70 eV (on the extended surface), ensuring the convergence of our energetics on the corner model to those of slab models involving no capping agent.

## Supplementary information

Supplementary Information

Description of Additional Supplementary Files

Supplementary Movie 1

Supplementary Movie 2

Supplementary Movie 3

## Data Availability

The data that support the findings of this study are available from the corresponding author upon reasonable request.
