# Supplementary material for: Atomistic insights into the nucleation and growth of platinum on palladium nanocrystals
Source: Nat Commun. 2021 Jun 2;12:3215. doi: 10.1038/s41467-021-23290-x (PMC8173021; doi:10.1038/s41467-021-23290-x)
Supplement: Supplementary file 1 — Supplementary Information [file 41467_2021_23290_MOESM1_ESM.pdf]

Supplementary Information for

Atomistic insights into the nucleation and growth of platinum on palladium nanocrystals

Wenpei Gao[1,2,#], Ahmed O. Elnabawy[3,⊥,#], Zachary D. Hood[4], Yifeng Shi[5], Xue Wang[5], Luke T. Roling[3,¶], Xiaoqing Pan[2,6,*], Manos Mavrikakis[3,*], Younan Xia[4,5,7,*], Miaofang Chi[8,*]

[1]Department of Materials Science and Engineering, North Carolina State University, Raleigh, NC 27695, United States

[2]Department of Materials Science and Engineering, University of California, Irvine, Irvine, California 92697, United States

[3]Department of Chemical and Biological Engineering, University of Wisconsin - Madison, Madison, Wisconsin 53706, United States

[4]School of Chemistry and Biochemistry, Georgia Institute of Technology, Atlanta, Georgia 30332, United States

[5]School of Chemical and Biomolecular Engineering, Georgia Institute of Technology, Atlanta, Georgia 30332, United States

[6]Department of Physics and Astronomy, University of California, Irvine, Irvine, California 92697, United States

[7]The Wallace H. Coulter Department of Biomedical Engineering, Georgia Institute of Technology and Emory University, Atlanta, Georgia 30332, United States

[8]Center for Nanophase Materials Sciences, Oak Ridge National Laboratory, Oak Ridge, Tennessee 37831, United States

⊥Current address: Chemical Engineering Department, Faculty of Engineering, Cairo University, Giza 12613, Egypt

¶Current address: Department of Chemical and Biological Engineering, Iowa State University, Ames, IA 50011, United States

Correspondence to: chim@ornl.gov, younan.xia@bme.gatech.edu, emavrikakis@wisc.edu, xiaoqingp@uci.edu,

[#]These two authors contributed equally to this work.

Supplemental Figures

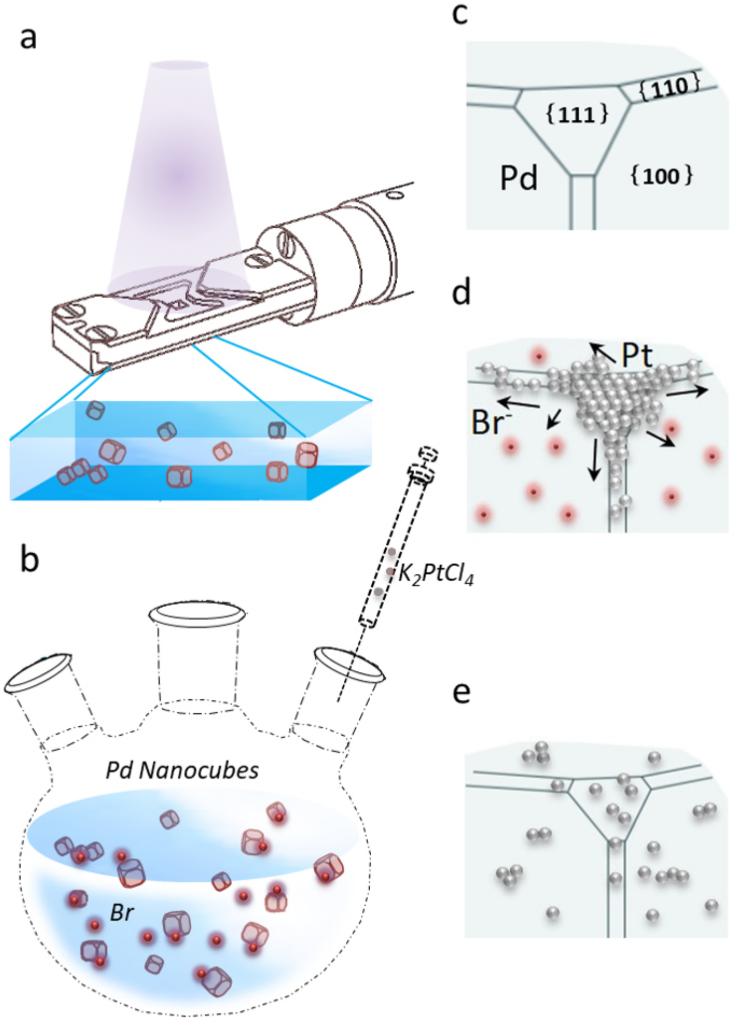

Supplementary Fig. 1. Comparison of the set-ups for LC-TEM and batch synthesis. a A schematic of the set-up for LC-TEM, where a mixture of Pd cubic seeds, Pt^{II} precursor, and KBr is sealed in a liquid cell consisting of two electron-transparent SiN windows. **b** A schematic illustration of the set-up typically used for the synthesis of Pd@Pt core-shell nanocubes in a batch reactor. **c** The three major facets on the surface of a Pd cubic seed. **d** The growth of a Pd cubic seed in the presence of Br^- capping on the {100} facets. **e** The growth of a Pd cubic seed in the absence of Br^- capping.

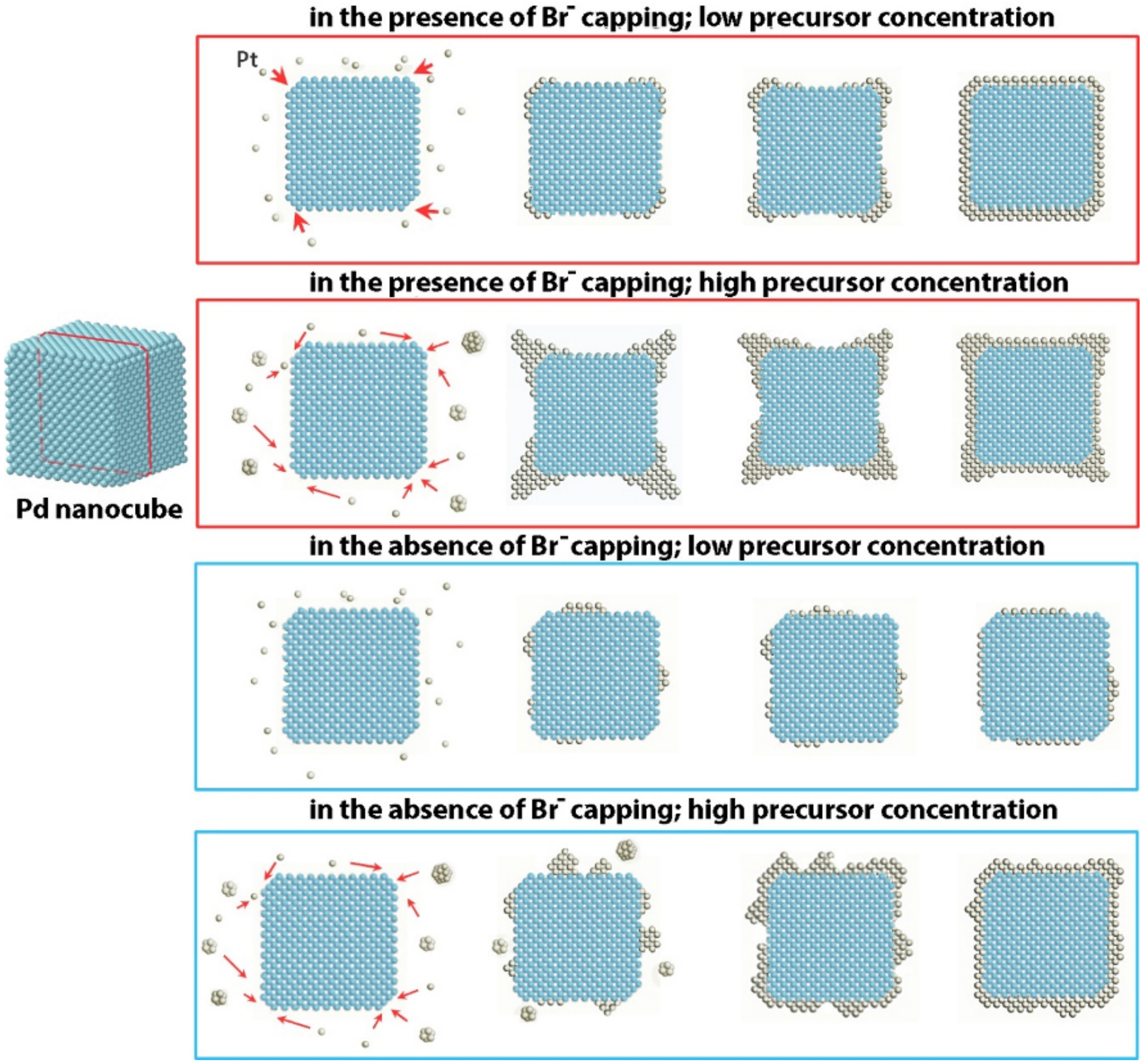

Supplementary Fig. 2. Atomic models showing the cross-section views of the core-shell particle formation processes, including the corresponding nucleation site, surface diffusion path, and growth pattern, under four different conditions evaluated in this study. The cross-section is defined by cutting along the red frame in the 3D cubic model.

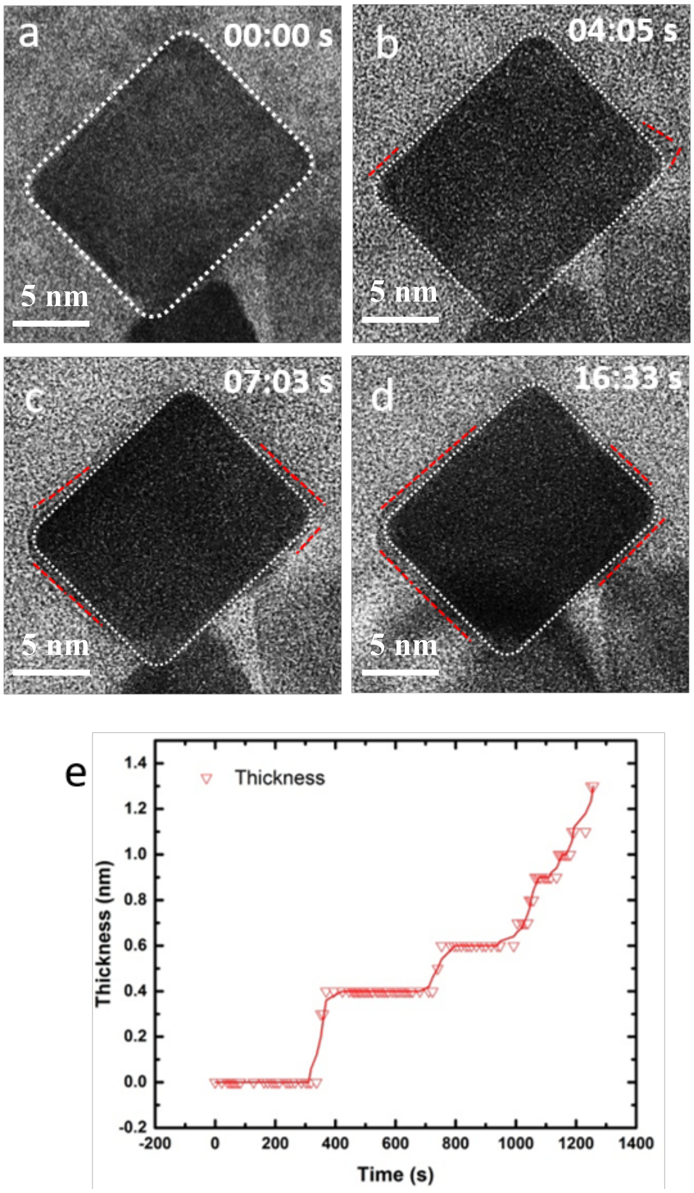

Supplementary Fig. 3. Growth of Pt on a Pd cubic seed at the low precursor concentration and in the presence of Br^- capping. a-d Snapshots taken from the video recorded during an *in situ* LC-TEM experiment. **e** Thickness of the Pt deposited on the corner as a function of time.

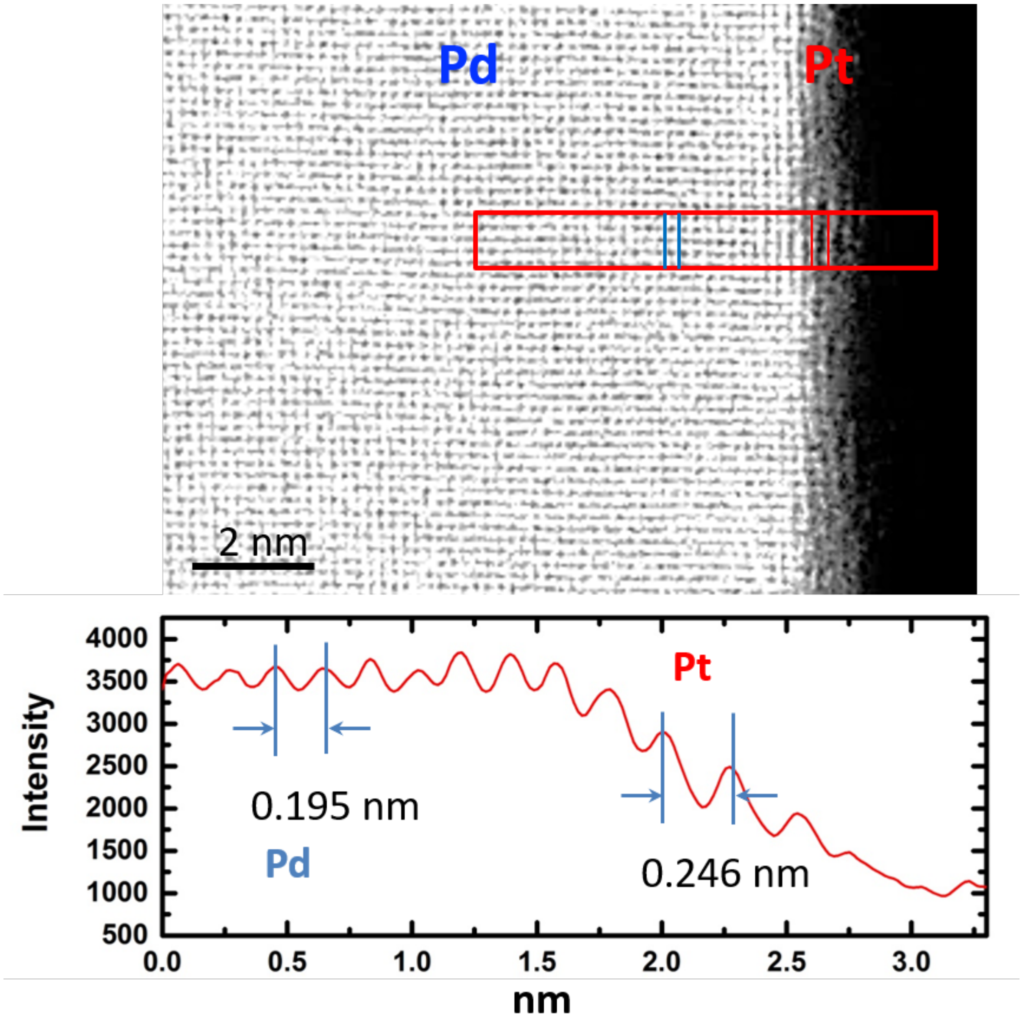

Supplementary Fig. 4. Lattice spacing between Pd(100) and Pt(100) planes. The newly formed Pt overlayer shows a larger spacing than that of Pd (0.246 nm vs. 0.195 nm), indicating expansion along the direction of surface normal due to the involvement of in-plane surface compression.

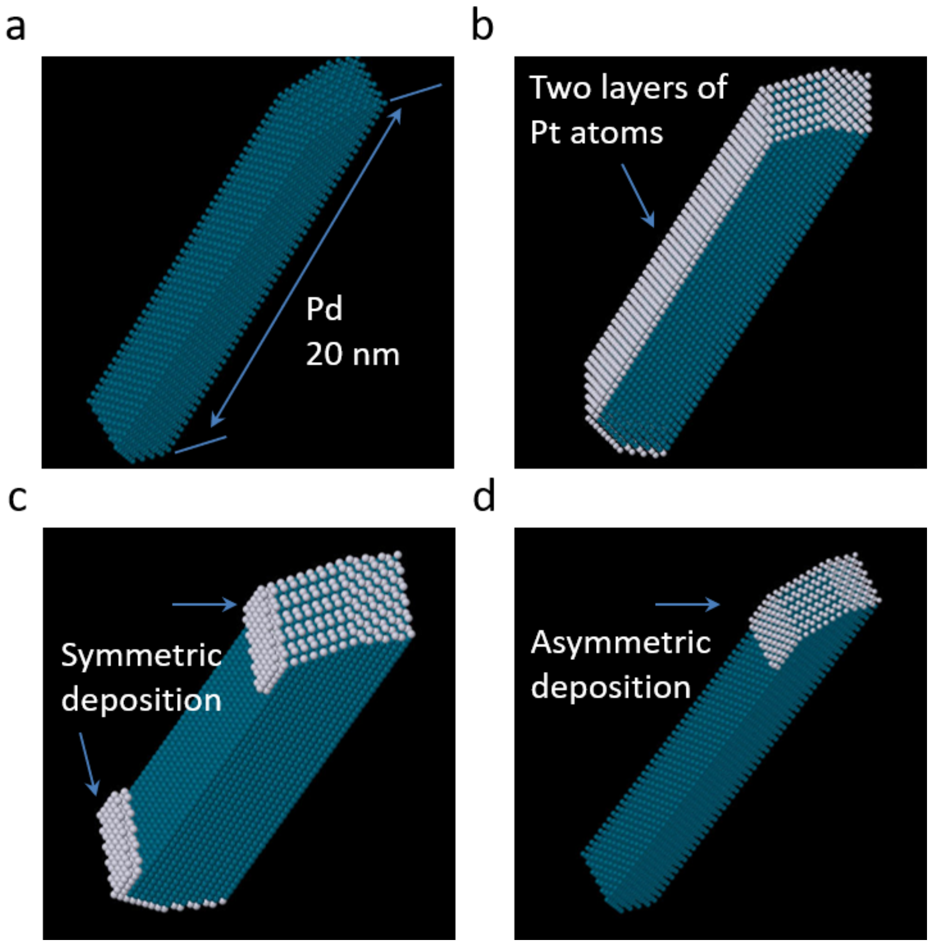

Supplementary Fig. 5. Atomic models of Pd and Pd@Pt nanocube for STEM image simulation. a. A portion of the Pd nanocube with truncated edges on both sides, the Pd nanocube is 20 nm measured in edge length. **b.** Two layers of Pt atoms form a complete shell covering the entire nanocube. **c.** Pd nanocube with two layers of Pt atoms symmetrically deposited on both edge regions. **d.** Pd nanocube with two layers of Pt atoms asymmetrically deposited only on one edge.

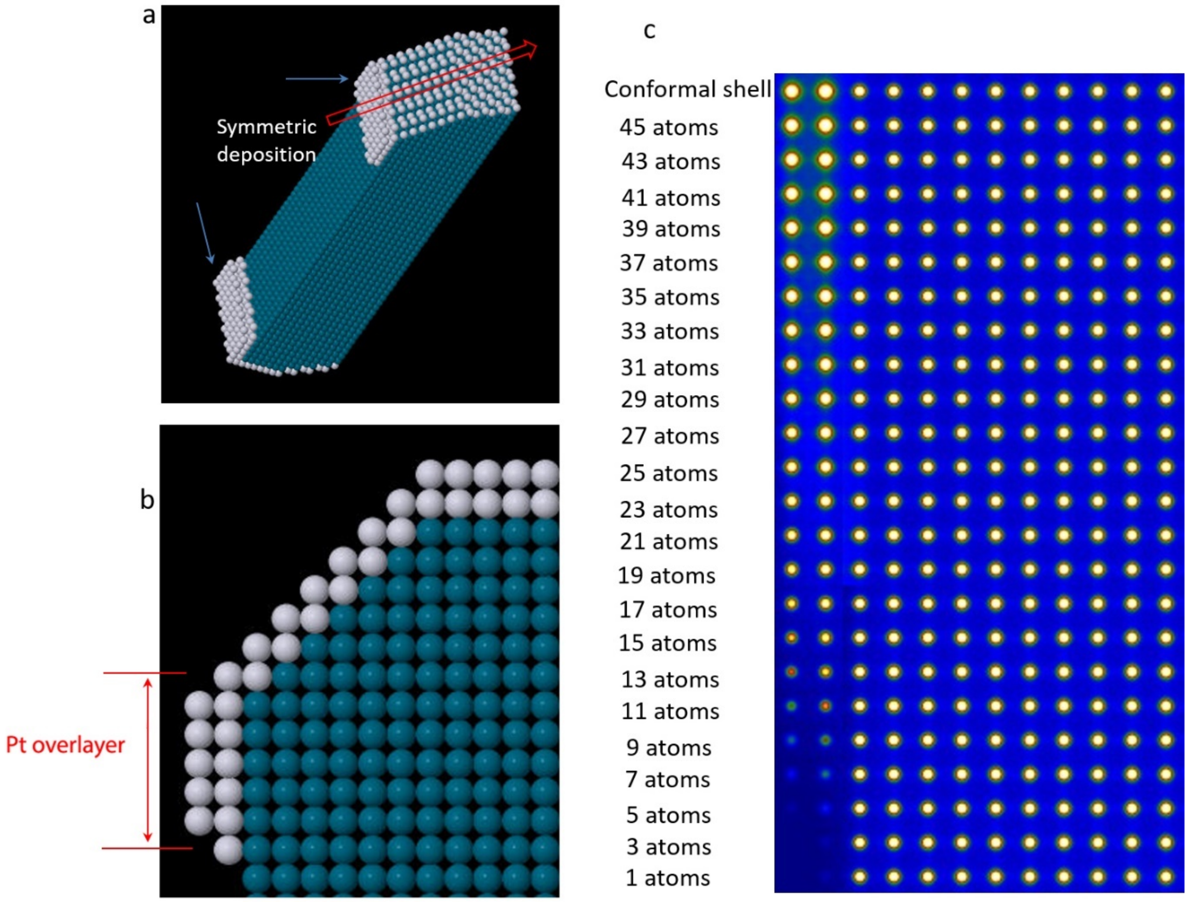

Supplementary Fig. 6. HAADF-STEM image from edge to the center of nanocube simulated using the atomic models with different coverages for the Pt overlayer under symmetric deposition. **a** A slab of the 3D model, the same as Supplementary Fig.5c, images from the boxed area is selected to show in **c**. **b** 2D projection of the atomic model in **a** from side view. The length of Pt layer deposited is defined by number of atoms included in the 'Pt overlayer'. **c** A table of simulated HAADF STEM images of Pd@Pt nanocubes with Pt layer of different lengths.

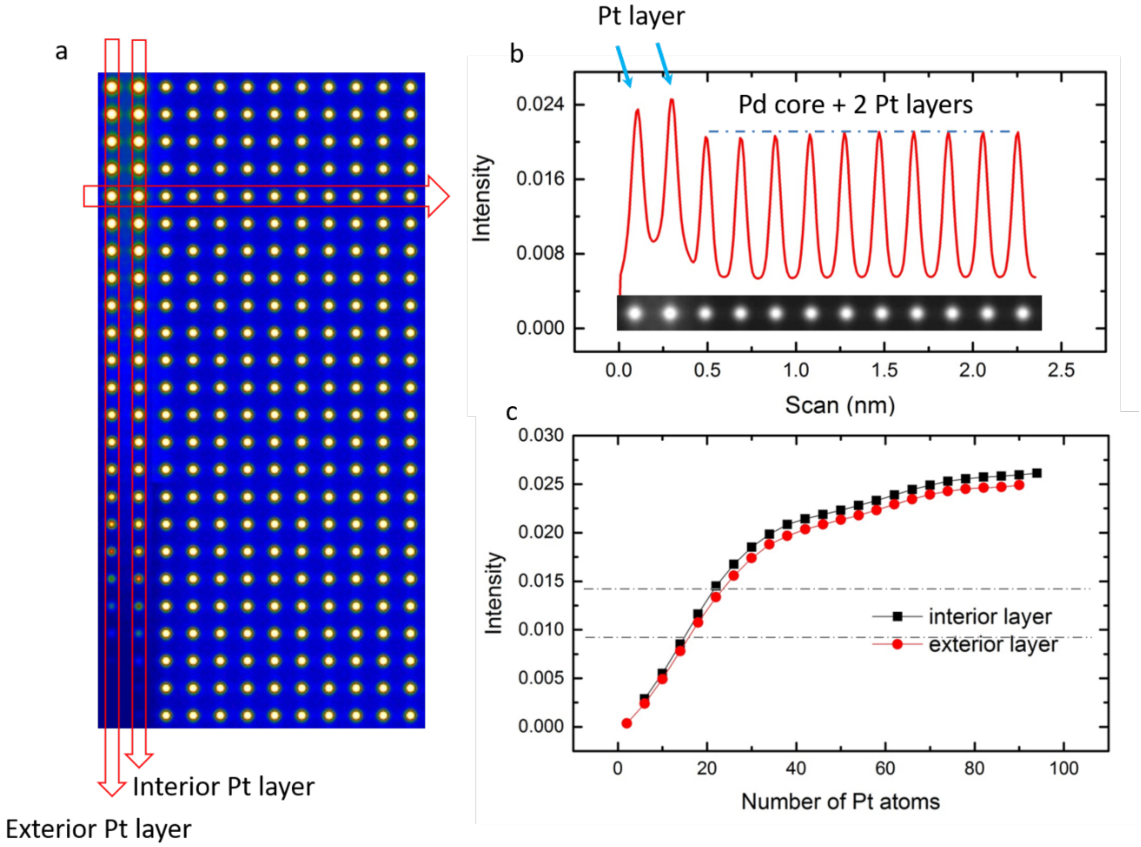

Supplementary Fig. 7. Intensity of HAADF-STEM image from simulation using the atomic model in Supplementary Fig. 6. a The vertical arrows mark the interior and exterior Pt layers of the nanocube. **b** An example of the intensity profile along the atom columns labeled by the horizontal red boxed arrow in **a**. Intensity from the Pt column is seen brighter than that from the core of the nanocube. **c** Plot of the intensity change with different lengths of Pt layers. The value (intensity) is measured by peak intensity.

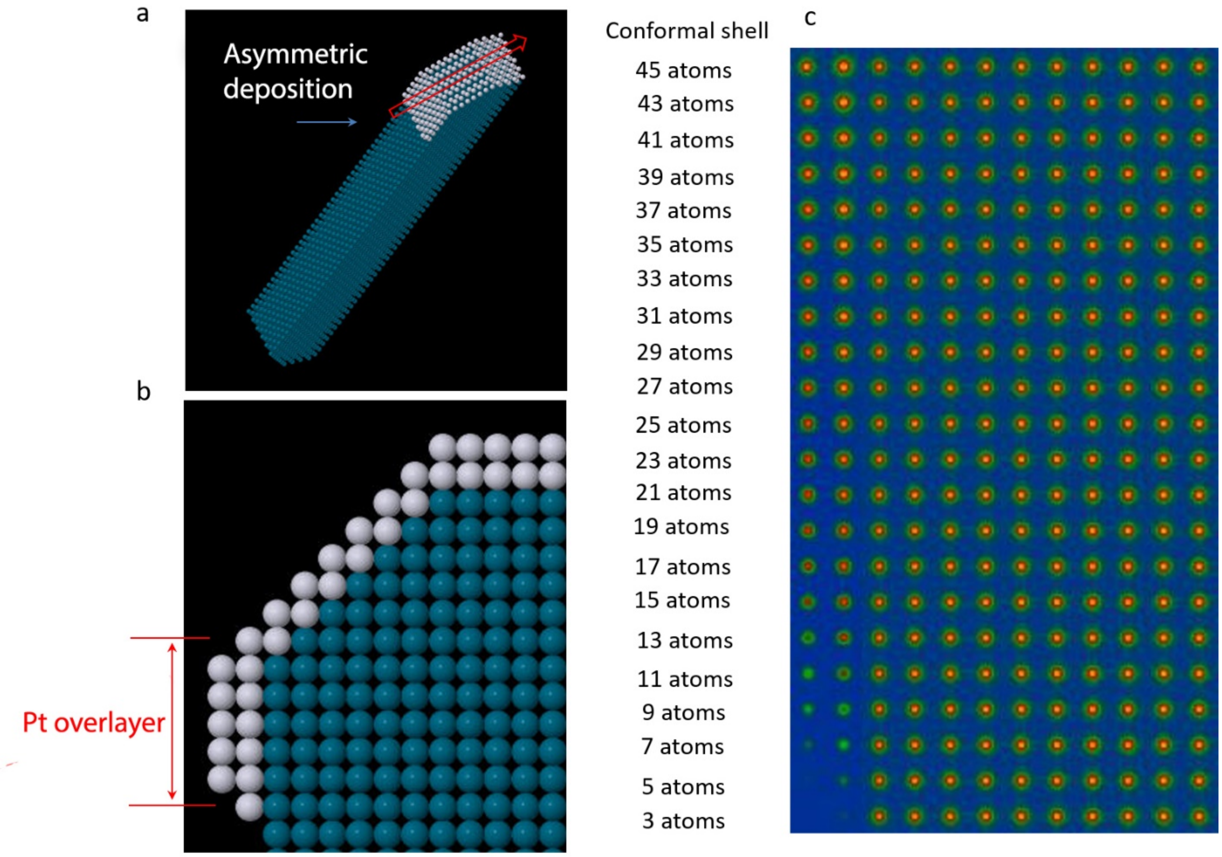

Supplementary Fig. 8. HAADF-STEM image from edge to the center of nanocube simulated using the atomic models with different coverages for the Pt overlayer under asymmetric deposition. a A slab of the 3D model, the same as Supplementary Fig.5d, images from the boxed area is selected to show in **c**. **b** 2D projection of the atomic model in **a** from side view. The length of Pt layer deposited is defined by number of atoms included in the 'Pt overlayer'. **c** A table of simulated HAADF STEM images of Pd@Pt nanocubes with Pt layer of different lengths.

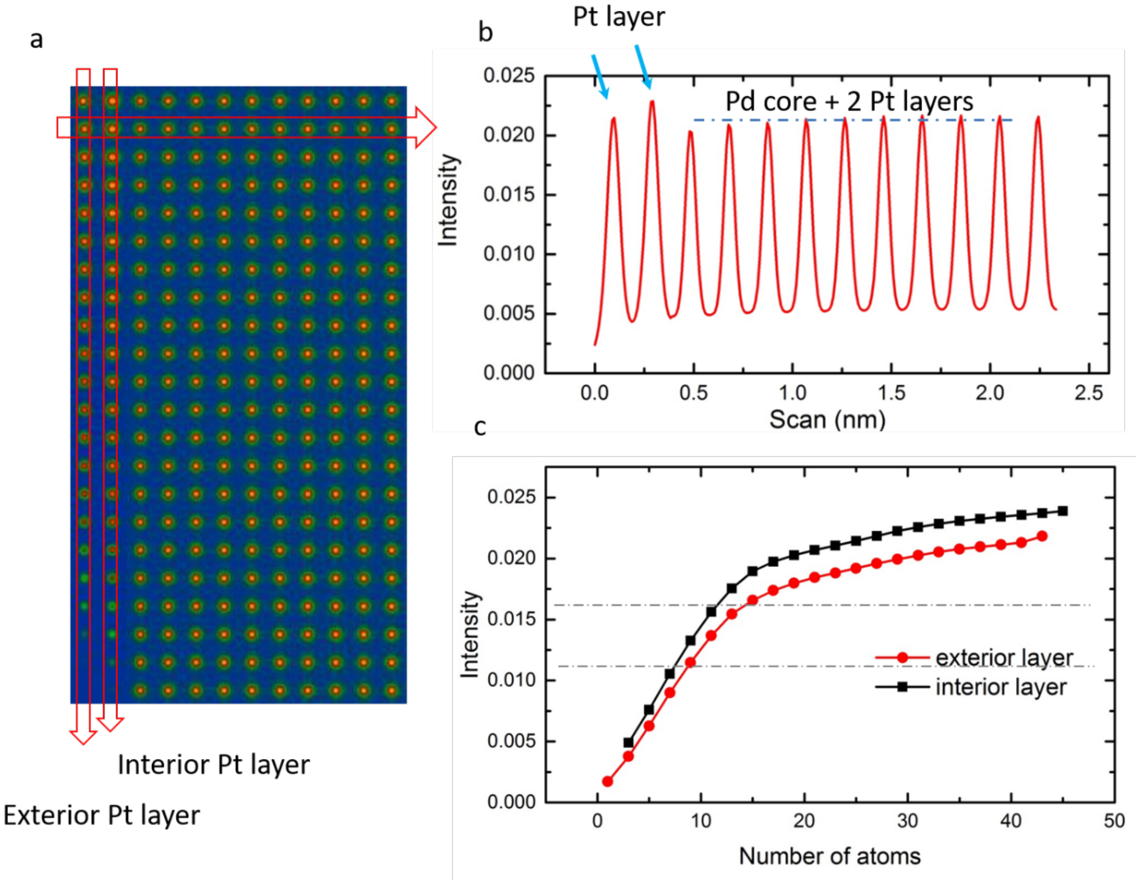

Supplementary Fig. 9. Intensity of HAADF-STEM image from simulation using the atomic model in Supplementary Fig. 8. a The vertical arrows mark the interior and exterior Pt layers of the nanocube. **b** An example of the intensity profile along the atom columns labeled by the horizontal red boxed arrow in **a**. Intensity from the Pt column is seen brighter than that from the core of the nanocube. **c** Plot of the intensity change with different lengths of Pt layers. The value (intensity) is measured by peak intensity.

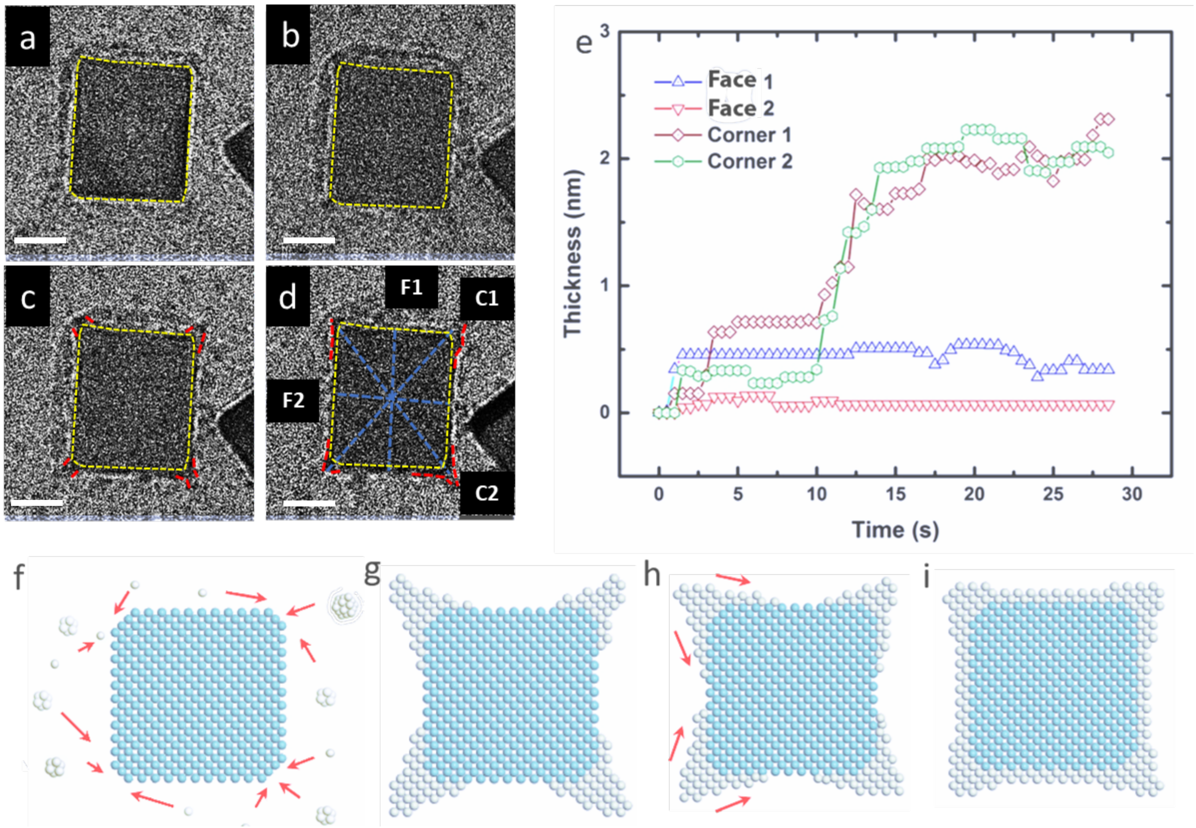

Supplementary Fig. 10. Growth of Pt on Pd cubic seeds capped by Br⁻ at a high concentration for the precursor. a-d Sequential TEM images recorded during an *in situ* experiment, showing the initial attachment of Pt cluster to the corner, followed by coalescence with Pd. The edge of the Pd nanocube is marked by yellow dashed line, and the contour of Pt deposited is highlighted by red lines. The C1 and C2 in **d** represent Corner 1 and Corner 2 in **e**; the F1 and F2 represent Face 1 and Face 2 in **e**. **e** Plots of the changes in Pt thickness on corners and faces as a function of time during *in situ* observation. **f-i** Atomic models illustrating the growth process in **a-d**. the models are the cross-section view of the same plane marked in supplementary Figure 2. Scale bar: 5nm.

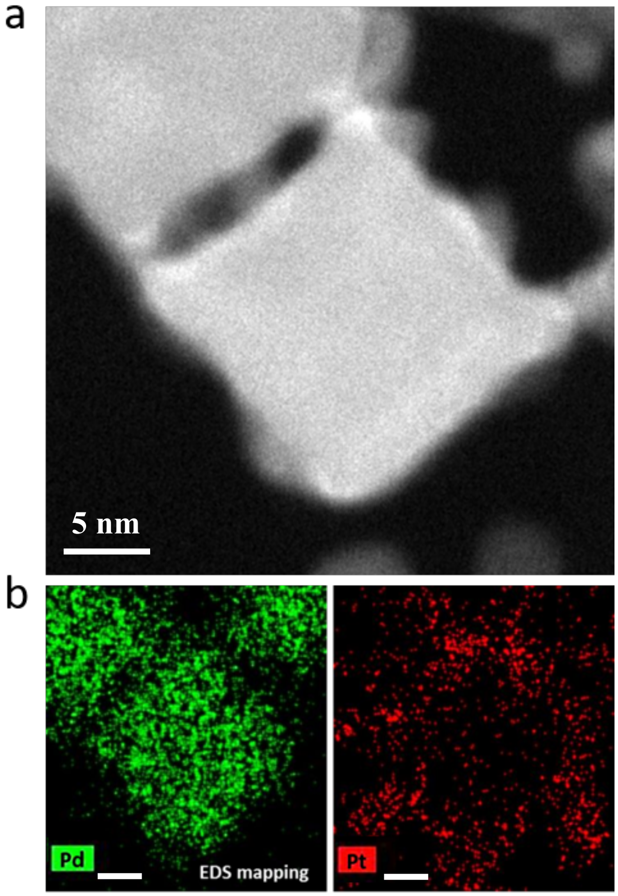

Supplementary Fig. 11. EDS mapping of a Pd@Pt core-shell nanocube formed at a relatively high concentration. **a** STEM Z-contrast image and **b** corresponding EDX maps of a typical nanocube formed at a relatively high precursor concentration and in the presence of Br^- capping, under the same condition as used in Fig. 3a. scale bar: 5nm.

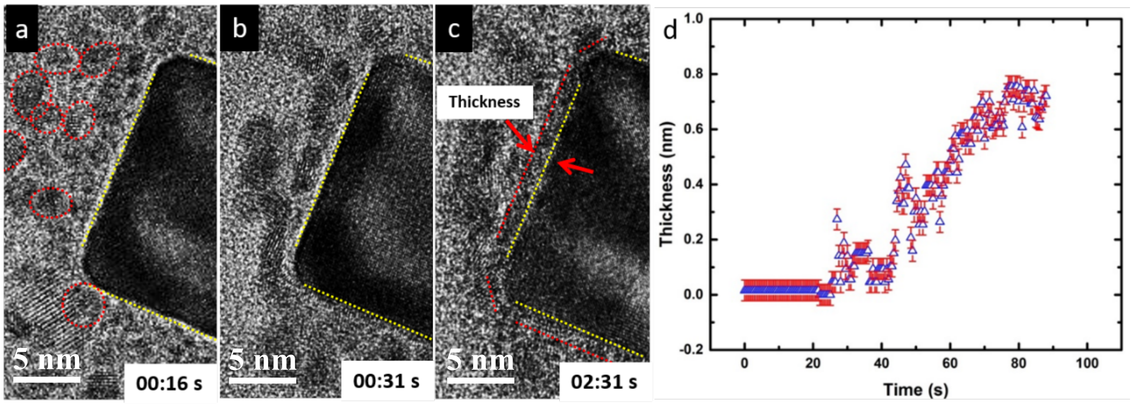

Supplementary Fig. 12. a-c *In situ* TEM images showing the formation of Pt clusters through self-nucleation at a high concentration for the precursor and in the absence of Br^-, followed by their attachment to and coalescence on the surface of the Pd cubic seed. **d** Plot of the thickness of Pt deposited on the face of the Pd seed as a function of time.

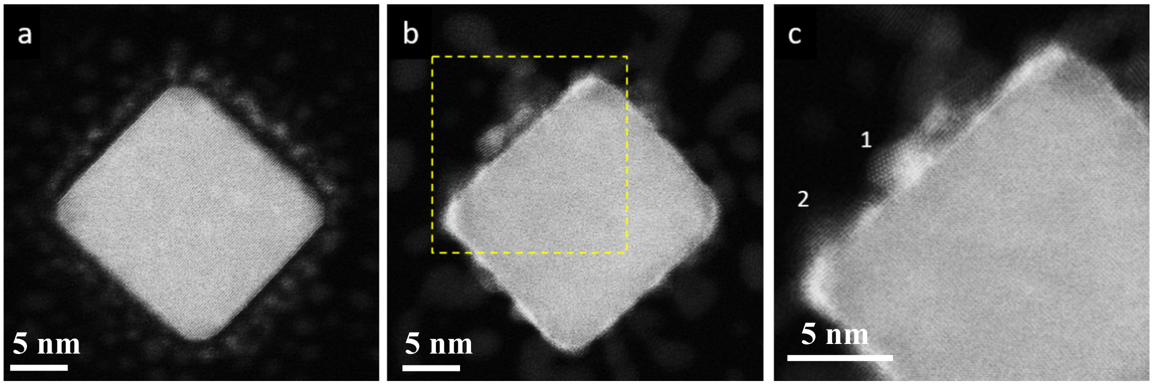

Supplementary Fig. 13. a An *ex situ* HAADF-STEM image of a Pd cubic seed surrounded by the Pt clusters formed through self-nucleation. **b** A Pd@Pt nanocube featuring the attached Pt clusters. The zoom-in image of the area in the yellow dashed box is shown in **c**. The Pt layers follows the atomic structure of the Pd seed. In **c**, the attached Pt cluster 1 is randomly orientated, while cluster 2 takes the favored orientation, suggesting the growth of Pt shell via the attachment and coalescence mechanism.

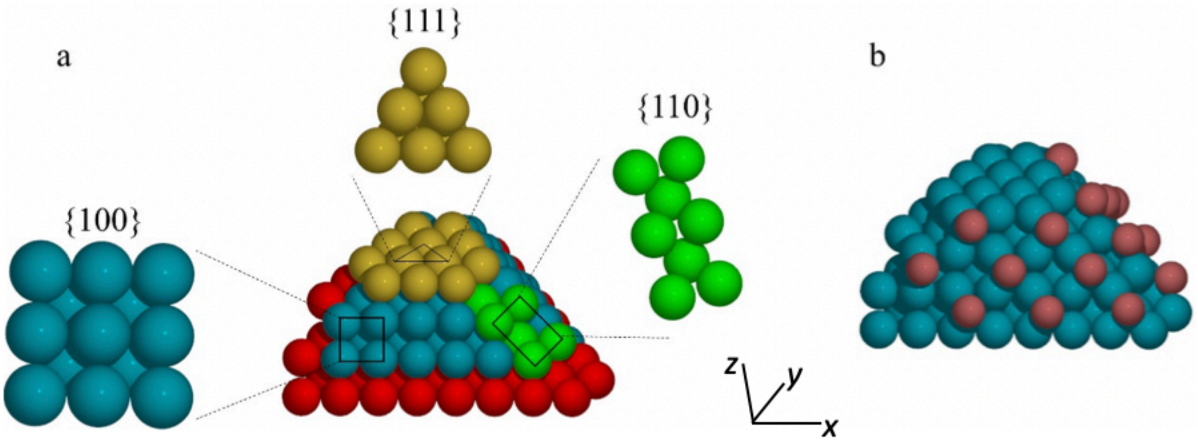

Supplementary Fig. 14. a The Pd cubic corner model. Blue spheres indicate the Pd atoms on the {100} facet, green spheres indicate the Pd atoms on the {110} facet, and yellow spheres indicate the Pd atoms on the {111} facet. Red spheres are Pd atoms fixed at bulk positions. **b** The most stable structure of the 0.5 ML Br adlayer on the {100} facet. The bottommost Br atoms (3 on either face; 6 in total) have their vertical coordinates fixed to avoid unphysical relaxations.

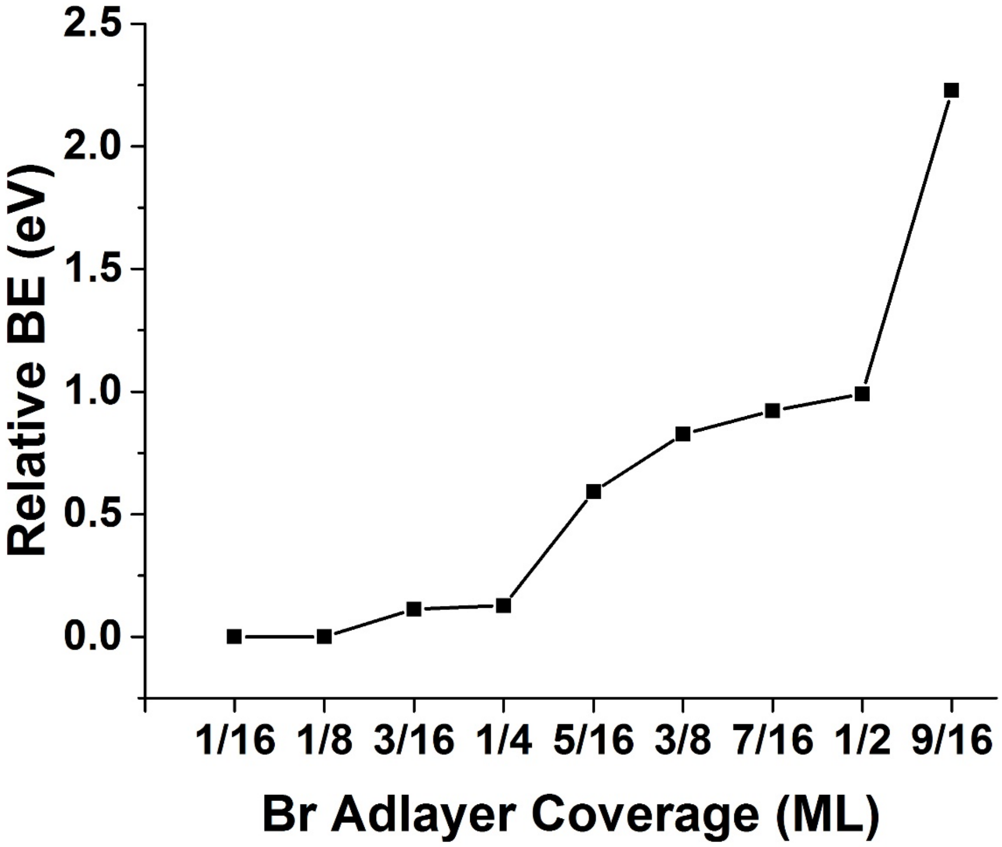

Supplementary Fig. 15. The relative binding energy (in eV) vs. the Br adlayer coverage (in ML). More positive relative binding energy (BE) indicates weaker binding.

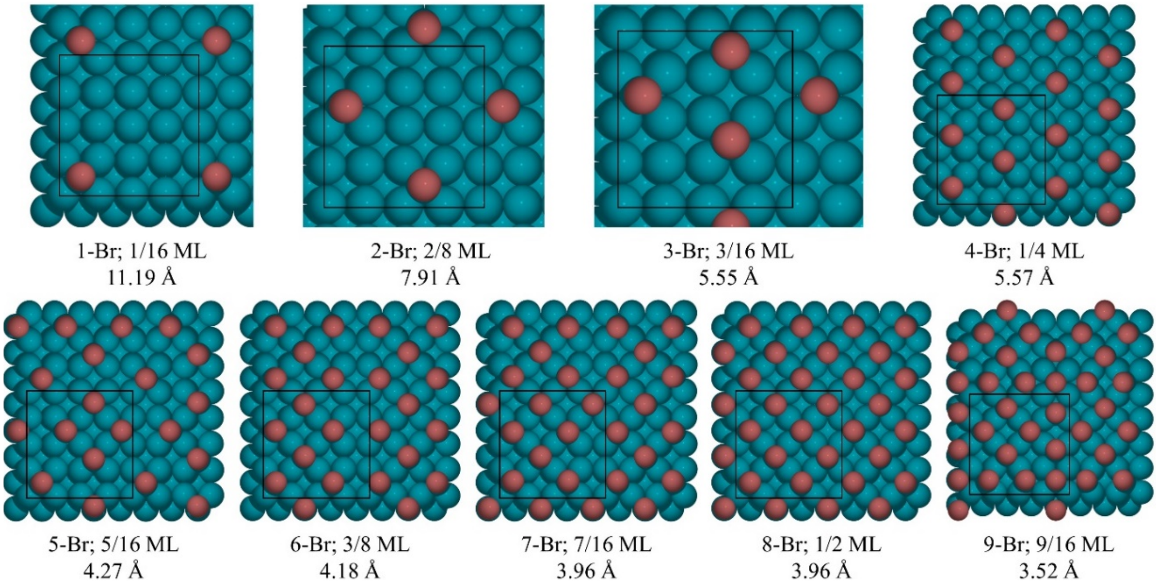

Supplementary Fig. 16. The Br adlayer structures up to 9/16 ML coverage at 1/16 ML increments on Pd(100), representing the faces of a Pd nanocube. In each inset, the black square marks the (4×4) unit cell; insets are enlarged at higher coverages to demonstrate patterns of the Br adlayer structure. Below each inset is the number of Br atoms within the unit cell, the coverage of the Br adlayer (in ML), and the shortest Br-Br distance (in Å). In the insets, blue and red spheres represent Pd and Br, respectively.

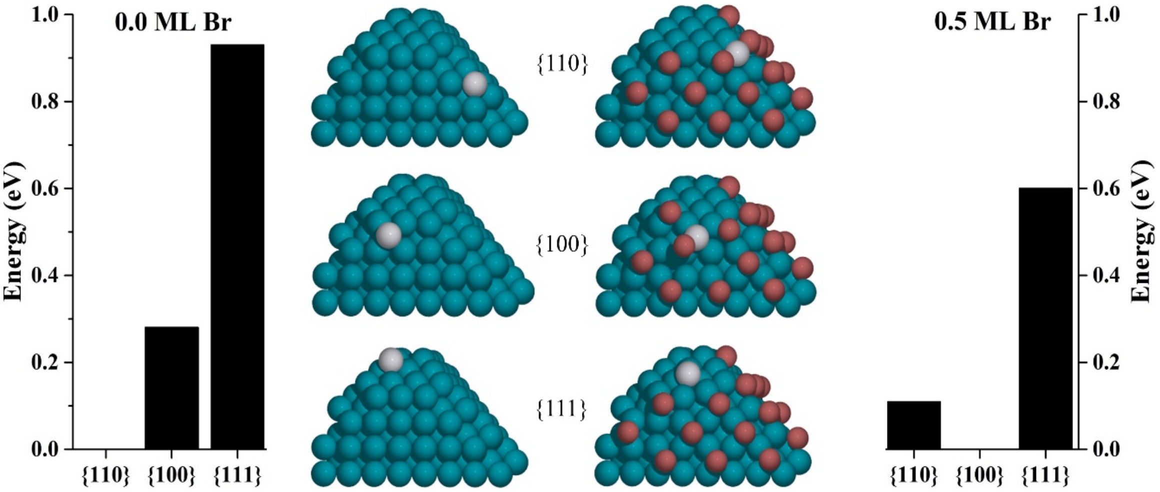

Supplementary Fig. 17. Relative energy of a Pt adatom on the three facets of the corner model, in the presence and absence of Br, respectively. In the insets, grey, blue, and red spheres represent Pt, Pd, and Br atoms, respectively.

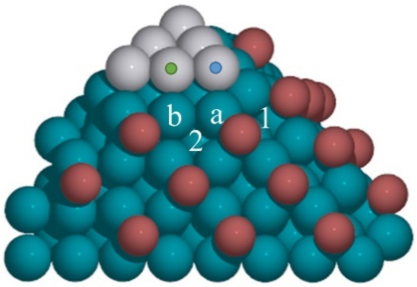

Path	Ea (eV)	ΔE (eV)
0→1	2.51 (1.41)	0.81 (0.41)
0→a→1	0.82 (0.81)	0.35 (0.30)
1→a→2	1.22 (0.75)	0.39 (0.37)
0→a→2	1.37 (1.18)	0.65 (0.51)
0→b→2	1.37 (0.88)	0.13 (0.62)

Supplementary Fig. 18. Diffusion of a Pt atom from a complete Pt layer on the {111} Pd corner. In the inset, hollow sites of interest are named numerically, while Pd atoms involved in the substitution mechanism are named alphabetically. The table lists the activation energies (*Ea*) and diffusion energies (*ΔE*) in eV for possible diffusion paths. In parentheses are the corresponding values in absence of Br. Each of the two diffusing Pt atoms is titled “0” in the table, with the color of the “0” corresponding to the color of the dot used to mark each of the Pt atoms. For example, the “0→a→1” substitution path describes the path in which the Pt atom marked with the blue dot substitutes and pushes Pd atom “a” to hollow site “1”. The “1→a→2” path refers to the back substitution of a Pd atom on hollow site “1” to push the Pt atom occupying position “a” to hollow site “2”. Grey, blue, and red spheres represent Pt, Pd, and Br atoms, respectively.

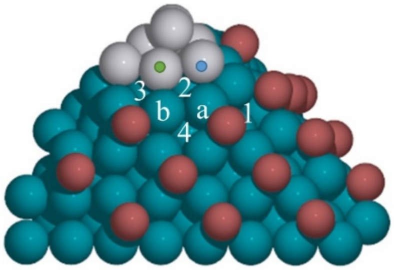

Path	Ea (eV)	ΔE (eV)
0→0→1	0.69 (0.47)	-0.24 (-0.50)
0→0→a→1	0.71 (0.44)	-0.60 (-0.72)
1→a→4	1.02 (0.89)	0.22 (0.47)
0→0→a→4	0.69 (0.57)	-0.30 (-0.51)
0→0→2	0.55 (0.50)	-0.12 (-0.26)
0→0→2	0.32 (0.58)	-0.12 (-0.26)
0→0→3	0.24 (0.35)	-0.67 (-0.32)
0→0→b→4	0.43 (0.23)	-0.99 (-0.60)

Supplementary Fig. 19. Diffusion of a Pt atom atop a complete Pt layer on the {111} Pd corner. In the inset, hollow sites of interest are named numerically, while Pd atoms involved in the substitution mechanism are named alphabetically. The table lists the activation energies (*Ea*) and diffusion energies (*ΔE*) in eV for possible substitution paths. In parentheses are the corresponding values in absence of Br. In the table, the Pt adatom is titled "0" in black, and each of the two diffusing Pt atoms underneath is titled "0", with the color of the "0" corresponding to the color of the dot used to mark each of these two Pt atoms. For example, the "0→0→a→1" substitution path describes the path in which the Pt adatom substitutes the Pt atom with the blue dot, which concertedly substitutes and pushes Pd atom "a" to hollow site "1". The "1→a→4" path refers to the back substitution of a Pd atom on hollow site "1" to push the Pt atom occupying position "a" to hollow site "4". The easiest substitution path "0→0→3" is depicted in Fig. 4, including states IS to FS-1, passing through TS-1. Grey, blue, and red spheres represent Pt, Pd, and Br atoms, respectively.

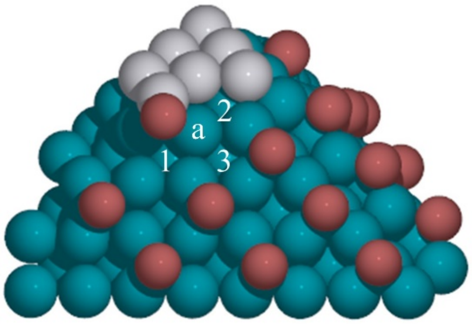

Path	Ea (eV)	ΔE (eV)
0→1	1.28 (1.05)	0.57 (0.07)
0→2	1.13 (0.85)	0.15 (0.06)
0→a→1	1.02 (1.06)	-0.13 (-0.26)
0→a→2	0.84 (1.14)	0.26 (-0.30)
0→a→3	0.80 (0.59)	-0.32 (-0.28)

Supplementary Fig. 20. Diffusion of a displaced Pt adatom along or away from the Pd-Pt interface. In the inset, hollow sites of interest are named numerically, while Pd atoms involved in the substitution mechanism are named alphabetically. The table lists the activation energies (*Ea*) and diffusion energies (*ΔE*) in eV for possible diffusion paths. In parentheses are the corresponding values in absence of Br. In the table, the Pt adatom is titled "0" in black, For example, the "0→1" path describes the Pt adatom hopping from its position in the inset to hollow site "1". The "0→a→1" path refers to the substitution of Pd atom marked "a" while pushing it to hollow site "1". The easier hopping path 0→2 is depicted in Fig. 4, including TS-2 and FS-2. Grey, blue, and red spheres represent Pt, Pd, and Br atoms, respectively.

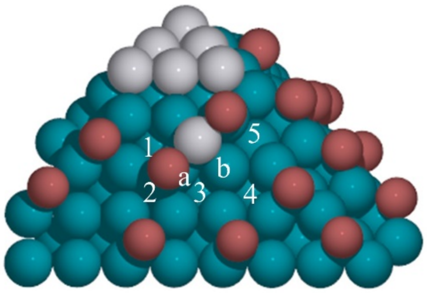

Hopping

Path	Ea (eV)	ΔE (eV)
0→1	0.74 (1.02)	0.74 (0.00)
0→3	0.79 (0.84)	0.31 (0.01)
0→5	0.73 (0.94)	0.05 (0.34)

Substitution

Path	Ea (eV)	ΔE (eV)
0→a→1	1.29 (1.23)	-0.02 (-0.38)
0→a→2	0.58 (0.58)	-0.34 (-0.37)
0→a→3	1.23 (1.15)	0.70 (-0.35)
0→b→3	1.41 (1.11)	0.65(-0.36)
0→b→4	0.93 (0.54)	0.68 (-0.36)
0→b→5	1.28 (0.63)	-0.06 (-0.09)

Supplementary Fig. 21. Diffusion of a Pt adatom away from the Pd-Pt interface. In the inset, hollow sites of interest are named numerically, while Pd atoms involved in the substitution mechanism are named alphabetically (atom "a" lies beneath a Br atom). The table lists the activation energies (*Ea*) and diffusion energies (*ΔE*) in eV for possible hopping and substitution paths. In parentheses are the corresponding values in absence of Br. In the table, the Pt adatom is titled "0" in black, For example, the "0→1" path describes the Pt adatom hopping from its position in the inset to hollow site "1". The "0→a→1" path refers to the substitution of Pd atom marked "a" while pushing it to hollow site "1". The easiest substitution path "0→a→2" is represented in Fig. 4, including FS-5 to FS-6 through TS-6. Grey, blue, and red spheres represent Pt, Pd, and Br atoms, respectively.

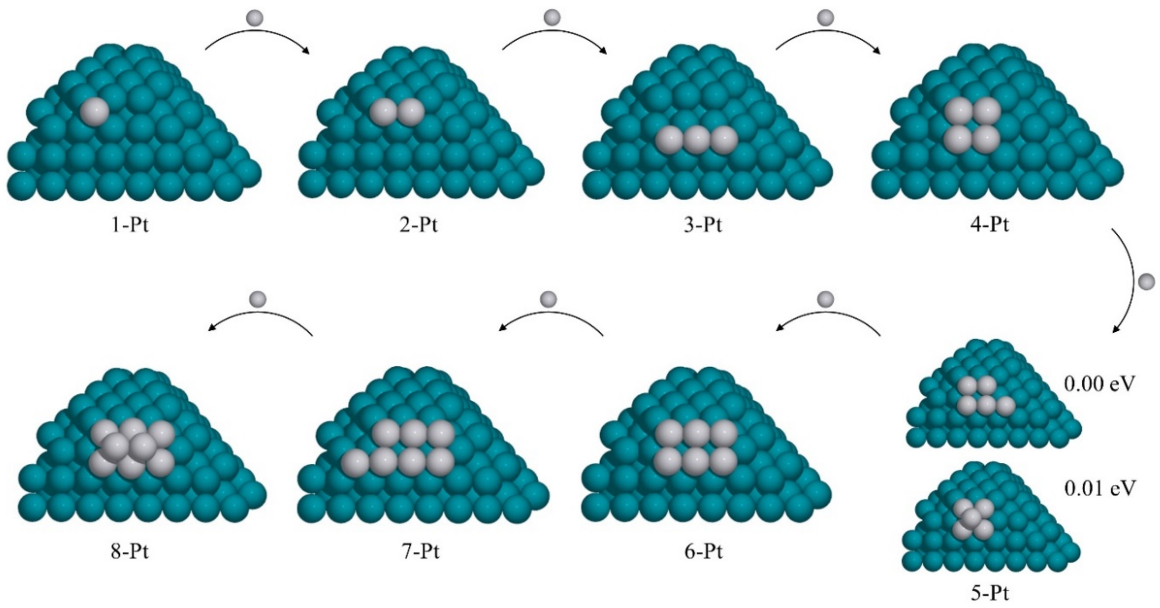

Supplementary Fig. 22. Most stable configurations of Pt atoms on the Pd(100) face. Labels below each inset represent the number of Pt atoms deposited. Insets are connected through curved arrows, each representing a single Pt atom addition to the previous structure. Two approximately isoenergetic configurations exist for the 5-Pt structure, with energies (in eV) relative to the more stable (top) configuration displayed to the right of each configuration. Grey and blue spheres represent Pt and Pd atoms, respectively.

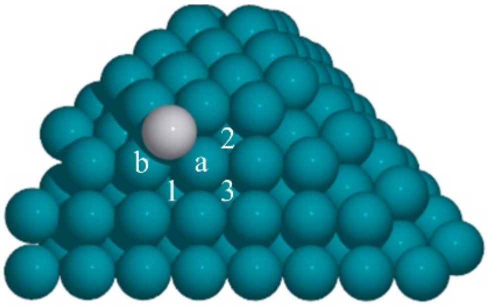

Hopping

Path	Ea (eV)	ΔE (eV)
0→1	1.00	0.09
0→2	0.94	0.01
2→3	1.01	0.13
3→1	1.19	0.09

Substitution

Path	Ea (eV)	ΔE (eV)
0→a→1	1.18	-0.26
0→a→2	1.19	-0.33
0→b→1	1.21	-0.30
0→a→3	0.66	-0.23

Supplementary Fig. 23. Diffusion of a single Pt atom via hopping or substitution. In the inset, hollow sites of interest are named numerically, while Pd atoms involved in the substitution mechanism are named alphabetically. The inset shows the Pt atom adsorbed in its most favorable site, titled "0" in the tables. The tables list the activation energies (*Ea*) and diffusion energies (*ΔE*) in eV for possible hopping and substitution paths. For example, the "0→1" hopping path describes the diffusion of the Pt atom from the site it occupies in the inset to the hollow site titled "1" through hopping over the bridge site between the two Pd atoms titled "a" and "b". On the other hand, the "0→a→1" substitution path describes the path in which the Pt atom (at the site it occupies in the inset) substitutes and pushes Pd atom "a" to hollow site "3". The easiest hopping path "0→2" and the easiest substitution path "0→a→3" are the paths depicted in Fig. 3a. Grey and blue spheres represent Pt and Pd atoms, respectively.

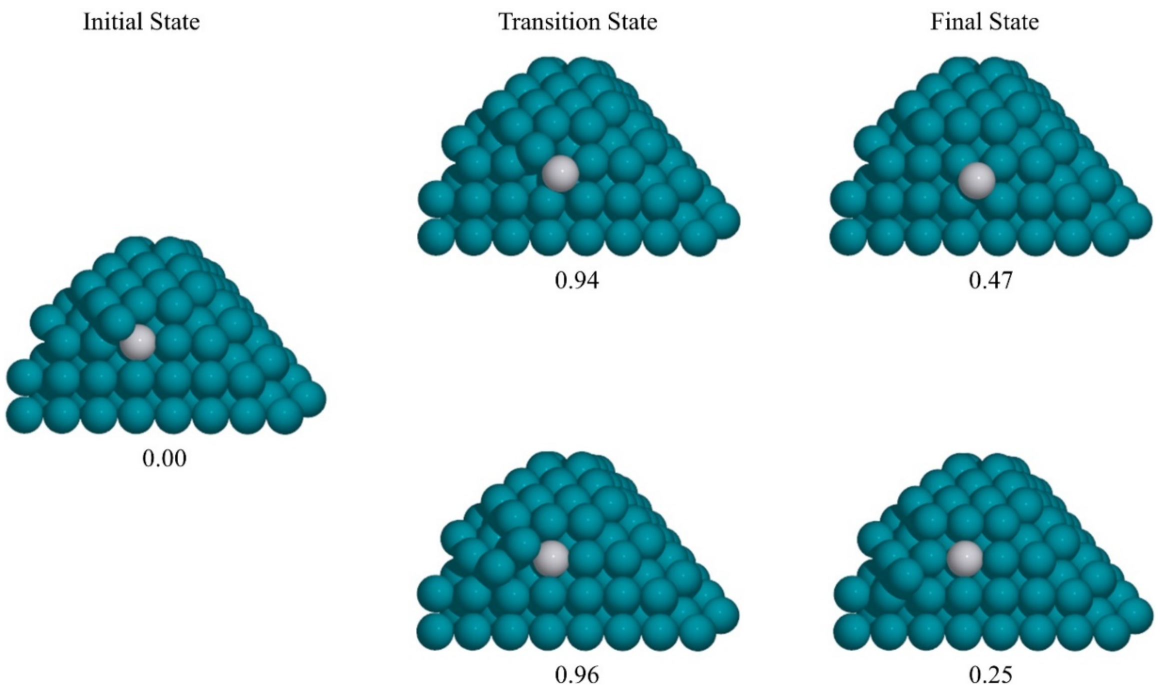

Supplementary Fig. 24. Substitution of a Pd atom into the {100} face replacing either a Pt atom (top row) or a Pd atom (bottom row). The leftmost column represents the initial state, the middle column represents the transition states, and the rightmost column represents the final states. Values below each structure are energies (in eV) relative to the initial state. Grey and blue spheres represent Pt and Pd, respectively.

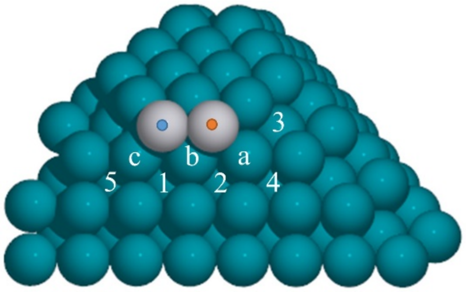

Hopping

Path	Ea (eV)	ΔE (eV)
0→1	0.84	0.45
0→2	0.82	0.48
0→3	1.36	0.76

Substitution

Path	Ea (eV)	ΔE (eV)
0→a→3	1.59	0.33
0→a→4	1.01	0.08
0→a→2	1.45	0.07
0→b→1	0.51	-0.26
0→b→2	0.46	-0.23
0→c→1	1.49	0.04
0→c→5	1.12	0.11

Supplementary Fig. 25. Diffusion of a Pt atom via hopping or substitution from a Pt dimer. In the inset, hollow sites of interest are named numerically, while Pd atoms involved in the substitution mechanism are named alphabetically. The inset shows the most stable configuration of the Pt dimer. Each Pt atom is titled "0" in the tables, with the color of the "0" corresponding to the color of the dot used to mark each of the Pt atoms. The tables list the activation energies (*Ea*) and diffusion energies (*ΔE*) in eV for possible hopping and substitution paths. For example, the "0→1" hopping path describes the diffusion of the Pt atom marked with the blue dot to the hollow site titled "1" through hopping over the bridge site between the two Pd atoms titled "b" and "c" in the inset. On the other hand, the "0→a→3" substitution path describes the path in which the Pt atom marked with the orange dot substitutes and pushes Pd atom "a" to hollow site "3". The easiest hopping path "0→2" and the easiest substitution path "0→b→2" are the paths depicted in Fig. 3b. Grey and blue spheres represent Pt and Pd atoms, respectively.

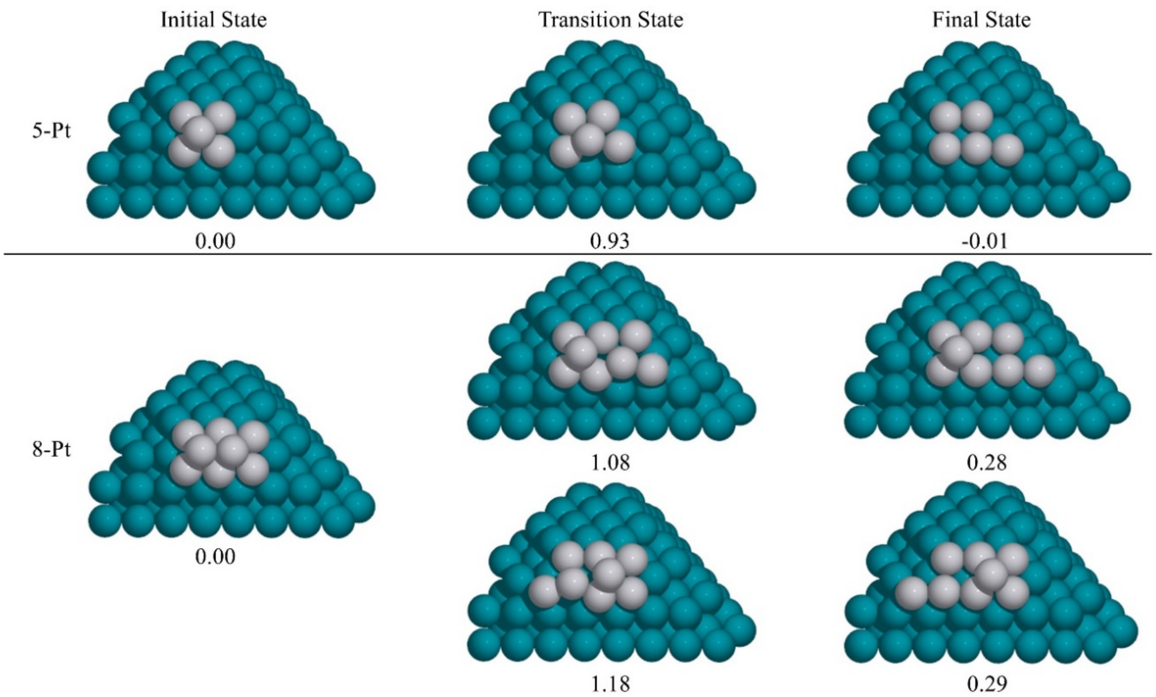

Supplementary Fig. 26. Breaking a Pt bilayer by means of substitution of a top Pt atom into an underlying Pt atom for a Pt Pentamer (5-Pt) and octamer (8-Pt). The 5-Pt case displays the substitution path connecting the two isoenergetic configurations shown in Supplementary Fig. T4. Two substitution paths are displayed for the 8-Pt case. The leftmost column represents the initial state, the middle column represents the transition states, and the rightmost column represents the final states. Values below each structure are energies (in eV) relative to the initial state. Grey and blue spheres represent Pt and Pd, respectively.

Supplemental Discussion

1. Another Case of Growth of Pt Layers on Pd Cube with Br^- Capping with Dilute Precursor

Another example of Pt growth on the Pd cubic nanoparticle is shown in Supplementary Fig. 3. The intact Pd nanoparticle at 0 s is shown in (a), with the edge of the nanoparticle being highlighted by the white dotted line. In (b), Pt starts to deposit on the two corners as indicated by the red outlines. At 7:03 s, in (c), the deposited Pt on the corners started to extend towards the two surfaces and continued until 16:33s. The measured thickness of the newly deposited Pt layer on the left corner indicated in (c) is shown in the plot in (e). Two rapid increases of 0.4 nm and 0.2 nm in thickness are revealed at the deposition time of ~320 seconds and 720 seconds respectively, corresponding to the deposition of two and one atomic layer of Pt, respectively. Comparing to the uniform growth of Pt shell via cluster attachment and diffusion, the growth rate of less than 1 pm/s is much slower.

While only two sides from the projection have Pt layers slowly grown, no obvious Pt deposition was seen on the rest of the areas. This inhomogeneous growth results from the inhomogeneous static liquid environment inside the static liquid cell. During *ex situ* synthesis, the solution is usually kept at an elevated temperature to provide a homogeneous growth condition with well mixing of the precursor and capping agent. However, our observation is still representative in demonstration of the growth of Pt on Pd with a much lower concentration of the Pt precursor.

In the HAADF image in Supplementary Fig. 4, the Pt layers deposited on the Pd surface has a larger lattice spacing of 0.246 nm in comparison with that from Pd of 0.195 nm.

2. Atomic Models and Image Simulation

Atomic models were built to quantitatively show the dimension and thickness of the deposited Pt layers along the depth direction. The initial model of Pd cubic nanoparticle was based on the atomic resolution HAADF images of Pd nanocubes. In Supplementary Fig. 5a, a quarter of the Pd cubic nanoparticle is shown along the Pd (100) orientation. A small Pd (111) facet can be seen on the corner with the dimension of 5-7 atomic spacing, therefore, in the atomic model, we built a Pd cubic nanoparticle with the size of 20 nm enclosed by {100} planes on the surface, {110} planes of 7 atomic spacing width on the edges and {111} small facets on corners, as shown in Supplementary Fig. 5b. The cubes could have extra rounding on corners (on the left corners in (b)) due to surface reconstruction, which is in accordance with the observed rounding at corners on the HAADF image. However, in the models we employed for image simulation, this rounding effect was not considered, because this should not have a key influence on the simulated image intensity in HAADF images.

Based on the initial model of Pd cubic nanoparticles, those with two Pt layers deposited on the surface were further built, with the variance of the length of the Pt layer along the beam direction. As shown in Supplementary Fig. 5, we employed two modes when building the atomic

model: symmetric deposition of Pt on both sides of the Pd cube as shown in Supplementary Fig. 5c, and deposition of Pt only on the top side of the Pd cube as in Supplementary Fig. 5d.

From the simulated image using multi-slice simulation (Zmult),[1] we extracted the atomic column image of the outermost Pt layer, the inner Pt layer and the Pd nanocube including the outermost Pd surface across the truncated edge and the flat Pd surface. As shown in Supplementary Fig 6. These images from the model of symmetric deposition with different Pt layer dimension are summarized in Supplementary Fig. 6c. The intensity profiles were then extracted from this summarized intensity table (Supplementary Fig. 6c and Fig. 7a). An exemplary intensity profile of the model with symmetrically deposited Pt layers of 39 atoms from each side is shown in Supplementary Fig. 6b. In total, there are 76 Pt atoms from the outermost layer and 78 Pt atoms from the inner Pt layer in this projection in this case, for comparison, there are 100 Pd atoms plus two layers of Pt on each side along this projection. As shown in the intensity profile, the intensity from Pt layers is higher than that from Pd. And even though the Pd profile includes the truncated edges, the intensity from the edge is not obviously weaker than that from the flat surface. This is because the intensity of HAADF image from heavy scatters like Pd and Pt will not be linear with the number of atoms after a few nm in thickness. This can be seen from the plot of intensity vs. number of Pt atoms in Supplementary Fig. 7c. In this plot, the number is counted by adding the Pt atoms from two sides from the model of symmetrical deposition. For example, the number of 20 in x coordinate corresponding to the Pt deposition of 10 atoms on each side. The intensity increases

linearly with the number of Pt atoms before 40, corresponding to the length of Pt of ~ 5 nm on each side.

Similarly, the intensity table and intensity profile from the model of single deposition of Pt were processed, extracted and plotted in Supplementary Fig. 8 and Fig. 9. In this model, again, we see that the relationship of intensity with the number of Pt is linear only below 20 Pt atoms. In comparison, we find the intensity of Pt is not significantly weak in this plot, comparing with that from the model with symmetric Pt deposition. This is because the depth of focus of the electron probe is 6-10 nm, which defines the resolution in depth direction within STEM. By comparing the intensity of Pt layer and Pd from experiment with those from image simulation, we estimated there are approximately 10 Pt atoms in the Pt layers shown in Fig. 2b, c in main text and in Supplementary Fig. 3.

3. Justification for the use of 0.5 ML coverage of Br on the Pd corner model

The Br adlayer structure was calculated at successive coverages of one through nine Br atoms corresponding to 1/16, 1/8, 3/16, 1/4, 5/16, 3/8, 7/16, 1/2 ML, and 9/16 ML. At each coverage, we calculate the relative binding energy (*BE*) of an nth Br atom on Pd(100) as compared to that of the (n-1)th Br atom as:

Relative $BE|n = (E_{slab+nBr} - E_{slab+(n-1)Br} - Ref) - (E_{slab+(n-1)Br} - E_{slab+(n-2)Br} - Ref)$

where, n is the number of Br atoms adsorbed on the surface, starting at 2 Br atoms; $E_{slab+nBr}$, $E_{slab+(n-1)Br}$, and $E_{slab+(n-2)Br}$ are the total energies of the slab with n, (n-1), and (n-2) adsorbed Br atoms,

respectively; and Ref is an arbitrary reference state for a Br atom. Notice that with the definition above, Ref cancels out, and therefore no choice for the reference state needs to be made. The relative *BE* at 1/16 ML (n=1) is set to zero. Supplementary Fig. 15 shows the relative BE plotted for 1/16 to 9/16 ML, at 1/16 ML increments. In general, the relative *BE* keeps climbing to more positive energies, which indicates weakened binding of Br to the surface as coverage increases. We could identify two regions of moderate *BE* climbing: the n=1-4 region (1/16-1/4 ML), and the n=5-8 region (5/16-1/2 ML). These two regions are connected via a jump of 0.46 eV. A much larger jump (of 1.24 eV) connects the n=8 to n=9 structures (1/2-9/16 ML). To understand these trends, we provide in Supplementary Fig. 16 the most stable structure of the Br adlayer at each coverage. These structures show that Br is organized in such a way as to maximize the inter-Br distances; a direct consequence of the well-known repulsive dipole-dipole lateral interactions(*36, 37*) of Br (and halogens) on solid surfaces (including transition metals). In our unit cell, up to 1/4 ML, the shortest Br-Br distance is more than 5.50 Å. This distance drops to 4.27 Å commensurately with the 0.46 eV jump in relative *BE* mentioned earlier. Moving to higher coverages, the shortest Br-Br distance keeps gradually shrinking, culminating to 1/2 ML coverage to yield the $(\sqrt{2}\times\sqrt{2})R45°$ phase typical of fcc {100} surfaces.(*38*) In this structure, Br adatoms are equidistant at 3.96 Å. Adding a single Br atom to this structure disrupts this ordered phase, and reduces the shortest Br-Br distance to merely 3.52 Å at 9/16 ML, commensurate with the aforementioned jump of 1.24 eV in *BE*, thus, more than doubling the relative BE from its value at 1/2 ML coverage. This massive repulsion indicates that 1/2 ML coverage is a reasonable coverage to assume on the cubic

faces. It is worth noting here that recent *ab initio* thermodynamics coupled with the Gibbs-Wulff shape model found that the {100} facet is stabilized by 1/2 ML Br coverage as Pd gets squared into a cubic crystal.[2] We thus conclude that 1/2 ML is a reasonable estimate of the coverage of the Br adlayer on the {100} facets of the Pd cubes.

4. Diffusion of Pt Atom

When a single Pt atom is adsorbed onto a hollow site on the Pd(100) face, the Pt atom can diffuse to other hollow sites through hopping over Pd-Pd bridge sites, or substitute an underlying Pd atom, pushing it to an adjacent Pd surface hollow site. The easiest hopping and substitution diffusion paths for both substitution and hopping are shown in Fig. 5a, while all paths studied are shown in Supplementary Fig. 23. Hopping is slightly endothermic (only by 0.01 eV for the mechanism shown in Fig. 5), but is highly activated with an energy barrier of 0.94 eV. In contrast, substitution is exothermic by -0.23 eV, and is activated with a smaller energy barrier of 0.66 eV. This result suggests the substitution mechanism is clearly preferred for the diffusion of a Pt adatom on the Pd(100) face as shown in Fig. 5a. In this path, the substituted Pd atom is displaced to an adjacent hollow site that is inaccessible to the Pt atom in a single hopping step. The diffusion energies calculated here agree well with our recent calculations on extended surface models [3,4]. Supplementary Fig. 24 shows that the substitution of the displaced Pd atom into either the embedded Pt atom or the Pd atom embedded adjacent to it, is difficult (*i.e.*, activated by more than 0.90 eV), suggesting the stability of the embedded Pt atom. This indicates that a single Pt atom would preferentially substitute into the Pd(100) face and remain embedded in the {100} face.

Since there is no guarantee that Pt deposits on the surface as single atoms, we also explore the case of a Pt dimer. In this case, the most facile diffusion paths of both hopping and substitution are shown in Fig. 5b (all pathways studied are shown in Supplementary Fig. 25). Unlike the Pt monomer, for which hopping was slightly endothermic, hopping of one Pt atom away from the other is substantially endothermic (+0.48 eV) and is activated by 0.82 eV. Substitution of one Pt atom in this case is exothermic (-0.23 eV) and is only activated by 0.46 eV, which is 0.20 eV easier than the substitution in the Pt monomer case. Therefore, substitution is also more favorable to hopping, even more so than in the case of an isolated Pt atom on Pd.

With a complete ML of Pt on the {111} Pd corners, we calculate the possible diffusion paths of Pt from this ML. The same diffusion paths in the hypothetical scenario, where the Br adlayer is absent from the cube faces, were also studied to isolate the effect of Br on Pt diffusion. Supplementary Fig. 18 depicts the various diffusion paths and their corresponding activation and diffusion energies. All Pt atomic diffusion paths are highly activated in the presence and absence of Br. In either case, the most difficult path is the direct hopping of a Pt atom into the {110} edge. By contrast, the easiest path – in either case – is the substitution of a Pd {100} atom by a Pt atom, with the Pd atom pushed to a hollow site on the {110} edge (activation barriers of 0.82 and 0.81 eV in presence and absence of Br, respectively). The back diffusion of this Pd atom – for the Pd atom to return to its original position while pushing the occupying Pt atom onto a hollow {100} site – is also highly activated (1.22 and 0.75 eV in the presence and absence of Br, respectively), but less activated than the remaining diffusion paths considered. These findings suggest that: 1)

the {110} edge likely plays an important role in the spillover of thin Pt layers from the corners of the nanocube, and 2) thin Pt layers are likely to remain on the corner and not diffuse to cover the cube, given the high barriers associated with extracting a Pt atom from an environment in which it is coordinated with other Pt atoms on the corner.

In reality, Pt forms protrusions at Pd corners before they diffuse to other facets as revealed by experiments (Fig. 1), a scenario which we model with the diffusion of a single Pt adatom on a Pt layer (shortened as Pt adatom/monolayer in this paper). The discussion of Fig. 4 in the main text has shown a low barrier for the substitution of the Pt adatom into the complete Pt layer beneath, together with a facile diffusion along the Pd-Pt interface, reaching FS-2. To continue this discussion, moving further, the Pt atom diffuses away from the Pd-Pt interface. The hopping mechanism takes the Pt atom to FS-3, via TS-4, which is highly activated by 1.46 eV. Alternatively, FS-2 can reconstruct to the less stable FS-4 (with no activation energy) to start a substitution path in which a Pd atom is pushed to the {110} hollow site (TS-5, representing 0.68 eV activation energy), followed by reverse substitution to arrive at FS-5. The following diffusion step is then the linear substitution from FS-5 to TS-6 and into FS-6 (see Supplementary Fig. 21) for a full description of all these paths). These calculations thus reveal the atomistic diffusion pathways during the formation of core-shell particles, providing information that are complementary to the dynamic structural and chemical evolutions obtained by *in situ* microscopy.

5. Growth of Pt on Pd Cube with Br^- Capping with High Concentration of Precursor

For Pd cubes capped by Br^-, when increasing the precursor concentration, Pt clusters first nucleate in the liquid phase nearby the cubes. While we cannot exclude the growth of Pt on Pd by monomer deposition, the attachment of small Pt clusters on to Pd cubes is more obvious and dominant, which leads to faster growth on corners, as recorded in Supplementary Movie 2 and Supplementary Fig. 10. As shown in the plot in Supplementary Fig. 10e, the change of thickness across the sides and corners of the cube (E1, E2, C1, C2 as labelled in (d)) indicates that at the time of 10 s, there is a fast increase of diagonal length on C1 and C2, this is from the attachment and coalescence of Pt nuclei on the corners, the result is the obvious overgrowth on the corners as seen in Supplementary Fig. 10c. Meanwhile, the surface of the cube did not see obvious growth. From this observation, we propose the growth model of Pt on Pd when the concentration of Pt precursor is high, as in Supplementary Fig. 10f-i. The growth is completed by preferential attachment of small Pt nuclei and possible Pt monomers on the corners, in the presence of Br^-, followed by subsequent extension along the surfaces, possibly by both further deposition and atom diffusion. In Fig. 3a in the main text and Supplementary Fig. 11, the final particle shows a concave Pt shell outside the Pd cube, as evidenced by both the contrast and the EDS map.

6. Growth of Pt on Pd Cube without Br^- Capping

As shown in the main text, without Br^- capping, growth of monolayer of Pt on Pd surface can be obtained using a very low concentration of Pt precursor in the synthesis solution, however, it is not possible to control the sites where preferential growth occurs. And *in situ* TEM observation

did not capture this monolayer growth process due to multiple factors including the insufficient resolution of imaging within liquid, particle motion in liquid, and deficiency of contrast to distinguish atoms from different elements in HRTEM.

When we increased the concentration of the Pt precursor (Supplementary Fig. 12), Pt clusters were also observed. Those Pt clusters first attached onto the surface of Pd nanocube, without strong adhesion as evidenced by the bright contrast between Pd and Pt clusters. After 20 s, Pt atoms diffused from the attached Pt clusters onto the Pd surfaces, resulting in the increase of the size of cubic nanoparticles. Along with this process, the Pt cluster rotates to form epitaxy with the Pd surface, fitted with Pt(100)[010]||Pd(100)[010], as shown by the continuous lattice in HREM image in Supplementary Fig. 12c, Fig. 13c and the *ex situ* atomic resolution STEM image in Fig. 3 in the main text. This epitaxy is driven by the strong interaction between Pd and Pt, as well as the similarity of atomic lattice of Pd and Pt. Such observation of Pt cluster into a growing shell implies that the growth can also take place via a non-classical growth mechanism where crystals grow via attachment, re-orientation and coalescence of smaller nanocrystals.

7. Different modes and stages of Pt growth on Pd

The switch of growth mode from deposition of Pt monomers to attachment of Pt clusters can be achieved by changing the concentration of the Pt precursor. The direct deposition of Pt atoms follows the Pd atomic lattice from the beginning. If the shell is obtained by the attachment of Pt clusters, the Pt-Pd epitaxy may also be achieved via Pt cluster rotation on the surface, which also

resulted in the orientation relationship of Pt(100)\Pd(100). In the *ex situ* STEM images, we captured different stages of the growth, including small clusters approaching the cube surface (Supplementary Fig. 12a), attaching onto the surface, coalescing with each other, and rotating and aligning with the Pd cube at the orientations of Pt(100) \ Pd(100) (Supplementary Fig. 12b-c).

Study of growth dynamics is made possible with high spatial resolution. The results show that with controllable environment inside the liquid cell, *in situ* TEM is able to reveal detailed mechanisms during the shell growth. The control of shell growth into desired morphology is via the use of low concentration of precursor and the existence of capping agent. The use of Br^- ions as capping agent on the Pd surface leads to preferential initial deposition on the corners of Pd cubes, followed by progressive deposition of Pt along the surface and possible atomic diffusion from corners to facets. The result is a uniform continuous Pt shell of a few atomic layers over the entire particle. Higher concentration of Pt precursor doesn't change the preferential growth site on corners, but leads to concave Pt shell due to the faster growth on corners than atom diffusion on to facets. Without capping agent, increasing the concentration of Pt precursor changes the growth from random Pt deposition on Pd surface to Pt cluster attachment on Pd followed by epitaxy and diffusion to form a continuous shell with high surface roughness. These different growth modes are summarized in Supplementary Fig. 2.

References

1. Zuo, J.-M.; Spence, J. C. H., *Electron Microdiffraction*. Plenum: New York, 1991.
2. Yoo, S.; Lee, J.; Delley, B.; Soon, A, *Physical Chemistry Chemical Physics* **2014**, 16, 34, 18570-18577
3. Roling, L. T.; Mavrikakis, M. *Nanoscale* **2017**, 9, 39, 15005-15017.
4. Zhang, L.; Roling, L. T.; Wang, X.; Vara, M.; Chi, M.; Liu, J.; Choi, S.-I.; Park, J.; Herron, J. A.; Xie, Z.; Mavrikakis, M.; Xia, Y. *Science* **2015**, 349, 6246, 412-416.
