# Supplementary material for: Atomistic insights into the nucleation and growth of platinum on palladium nanocrystals
Source: Nat Commun. 2021 Jun 2;12:3215. doi: 10.1038/s41467-021-23290-x (PMC8173021; doi:10.1038/s41467-021-23290-x)
Supplement: Supplementary file 2 — Description of Additional Supplementary Files [file 41467_2021_23290_MOESM2_ESM.pdf]

Description of Additional Supplementary Files

File Name: Supplementary Movie 1
Description: *In situ* TEM video of the nucleation and growth processes of Pt on Pd cubic seed in the presence of Br^- ions at a low concentration of Pt^{II} precursor (0.015 mM).

File Name: Supplementary Movie 2
Description: Deposition of Pt on Pd cubic seed in the presence of Br^- ions at a high concentration of Pt^{II} precursor (1.5 mM).

File Name: Supplementary Movie 3
Description: Growth of Pt shell on Pd cubic seed in the absence of Br^- ions at a high concentration of Pt^{II} precursor.
